# High‐Efficiency Acousto‐Optic Modulation on Non‐Suspended Thin‐Film Lithium Tantalate

**DOI:** 10.1002/nap2.70275

**Published:** 2026-08-28

**Authors:** Weiran Zhou, Chengli Wang, Xuqiang Wang, Bowen Chen, Jiachen Cai, Tianyao Yang, Dongchen Sui, Xinjian Ke, Yang Chen, Xudong Wang, Ailun Yi, Shibin Zhang, Chengjie Zuo, Xin Ou

**Affiliations:** ^1^ School of Integrated Circuits University of Science and Technology of China Hefei China; ^2^ State Key Laboratory of Materials for Integrated Circuits Shanghai Institute of Microsystem and Information Technology Chinese Academy of Sciences Shanghai China; ^3^ Center of Materials Science and Optoelectronics Engineering University of Chinese Academy of Sciences Beijing China; ^4^ Shanghai Novel Si Integration Technology Shanghai China

## Abstract

Acousto‐optic (AO) interactions provide a powerful interface between the microwave and optical domains, enabling optical switching, non‐reciprocal propagation, and efficient microwave‐to‐optical transduction. Here, we demonstrate high‐efficiency AO modulation on a non‐suspended lithium‐tantalate‐on‐insulator (LTOI) platform. LTOI combines intrinsically low birefringence, a high optical‐damage threshold, strong electro‐optic and Kerr nonlinearities, and favorable acoustic properties. Our Mach–Zehnder interferometers achieve $V_{\pi }L=0.68$ V⋅cm, while racetrack resonators reach $V_{\pi }L=0.022$ V⋅cm, enabling microwave‐to‐optical conversion without suspended structures. Measurements across six in‐plane propagation directions identify propagation along the crystal *Z* axis as the most efficient tested configuration, where the higher‐order R1 mode produces the strongest MZI response. Temperature‐dependent acoustic measurements and continuous RF‐driven optical testing further demonstrate material‐related operating stability. These results establish non‐suspended LTOI as a robust platform for integrated acousto‐optics with broad applications in communications, signal processing, and quantum information technologies.

## Graphical abstract

We demonstrate high‐efficiency acousto‐optic modulation on a non‐suspended thin‐film lithium tantalate (LTOI) platform. By engineering acoustic modes within high‐Q resonators, the devices achieve low half‐wave voltage‐length products. This work establishes LTOI as a robust, scalable solution for integrated microwave‐photonics, combining strong piezoelectricity with industrial manufacturing maturity.

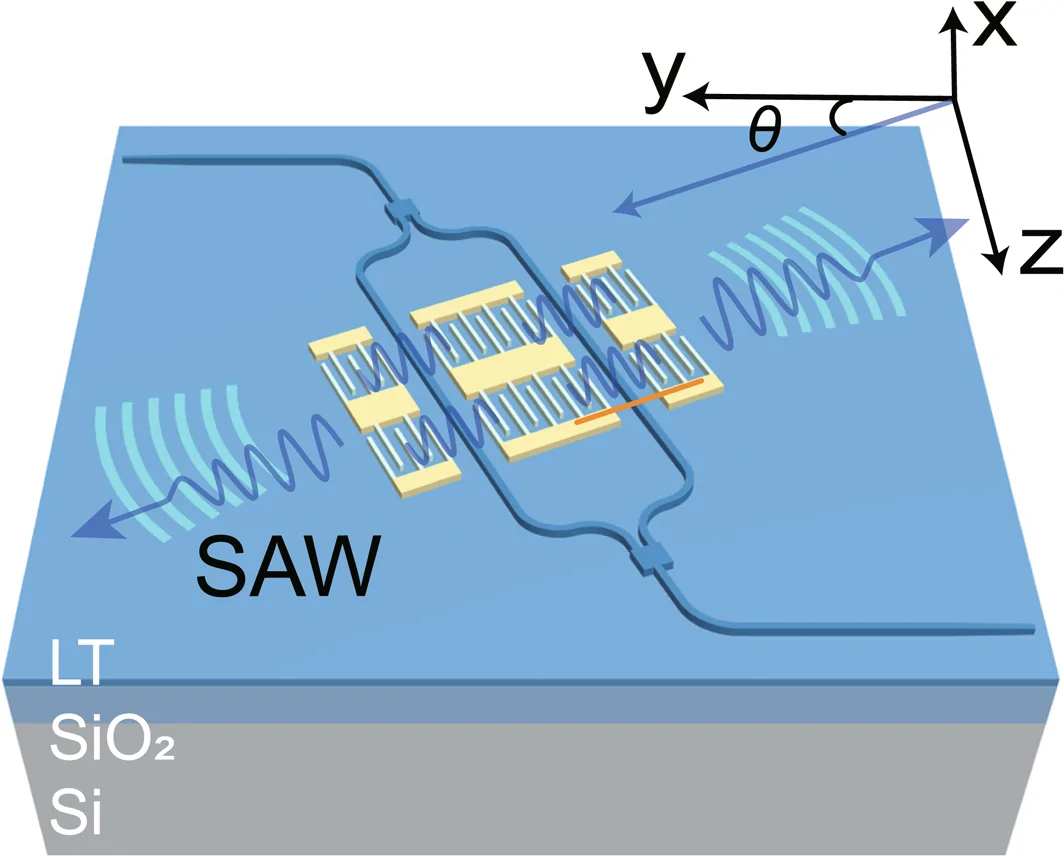

## 1 Introduction

Acousto‐optic (AO) interactions provide a versatile interface between microwave and optical domains, enabling functionalities that are otherwise challenging to achieve in integrated photonics. In particular, they allow for optical switching with high extinction ratios, as well as unique opportunities for non‐reciprocal light propagation, broadband spectral filtering, and highly efficient microwave‐to‐optical transduction [1, 2, 3, 4, 5]. To translate these opportunities into complex and large‐scale photonic circuits, it is crucial to realize efficient AO modulation on scalable and stable integrated platforms. Thin‐film piezoelectric materials such as AlN, GaAs, and TFLN have been widely explored for integrated AOs, where strong mechanical wave excitation and effective coupling with nanophotonic circuits were demonstrated [6, 7, 8, 9, 10, 11, 12, 13]. Among these, TFLN is regarded as a promising platform for AO interaction owing to its large electro‐optic coefficient, wide optical transparency window, low optical loss, and strong piezoelectric response enabling efficient acoustic excitation [14, 15, 16, 17, 18]. Nevertheless, practical implementations can be influenced by photorefractive effects and thermal drift, which affect device stability especially under high optical intensities [18]. Lithium tantalate (LT) provides a complementary combination of low birefringence, high optical‐damage threshold, strong electro‐optic and Kerr nonlinearities, and favorable acoustic properties [19, 20, 21, 22, 23, 24, 25]. Its reported temperature coefficients of frequency are generally smaller in magnitude than those of LN [26, 27]. LT is also established in commercial surface‐acoustic‐wave technology and benefits from a mature piezoelectric‐on‐insulator manufacturing ecosystem [28, 29, 30, 31].

Here, we demonstrate high‐efficiency AO modulation on LTOI and exploit the anisotropy of acoustic modes to achieve enhanced acoustic quality factors and efficient AO modulation. Our devices exhibit $V_{\pi }L$ values of 0.68 V⋅cm in Mach–Zehnder interferometers and 0.022 V⋅cm in racetrack resonators, the latter further enabling direct microwave‐to‐optical conversion without requiring suspended structures. We also evaluate LT‐related thermal‐frequency stability and long‐term optical‐power stability under continuous operation. These results establish LTOI as a practical and stable platform for high‐performance AO modulation, paving the way toward stable and multifunctional microwave‐photonic circuits and systems.

## 2 Device Design and Fabrication

The devices were fabricated on an LTOI wafer comprising a 600‐nm X‐cut LT film, a 4.7‐μm buried oxide layer, and a 525‐μm silicon substrate. Waveguides were defined by electron‐beam lithography and etched to a depth of 300 nm using $Ar^{+}$ ion‐beam etching at an incidence angle of 70°; the waveguide width was 1.2 μm. For the sub‐gigahertz MZI devices, the IDT wavelength was 6 μm, the electrode width was 1.5 μm, and 30 finger pairs were used. The IDTs and reflectors were formed by lift‐off of a 10/80‐nm Cr/Au bilayer, after which the chips were diced and assembled for optical and electrical coupling.

Figure 1a shows the AO MZI architecture. Light enters through an inverse taper, while a ground–signal–ground probe drives the IDT. The SAW propagates across the two MZI arms and is confined by acoustic reflectors. When interacting with the waveguide, the guided optical field—typically the fundamental TE mode—can be modulated through the electro‐optic, photoelastic, and moving‐boundary effects [32]. The arm separation is an odd multiple of half the acoustic wavelength, producing a π acoustic‐phase difference and push–pull phase modulation.

**FIGURE 1:**
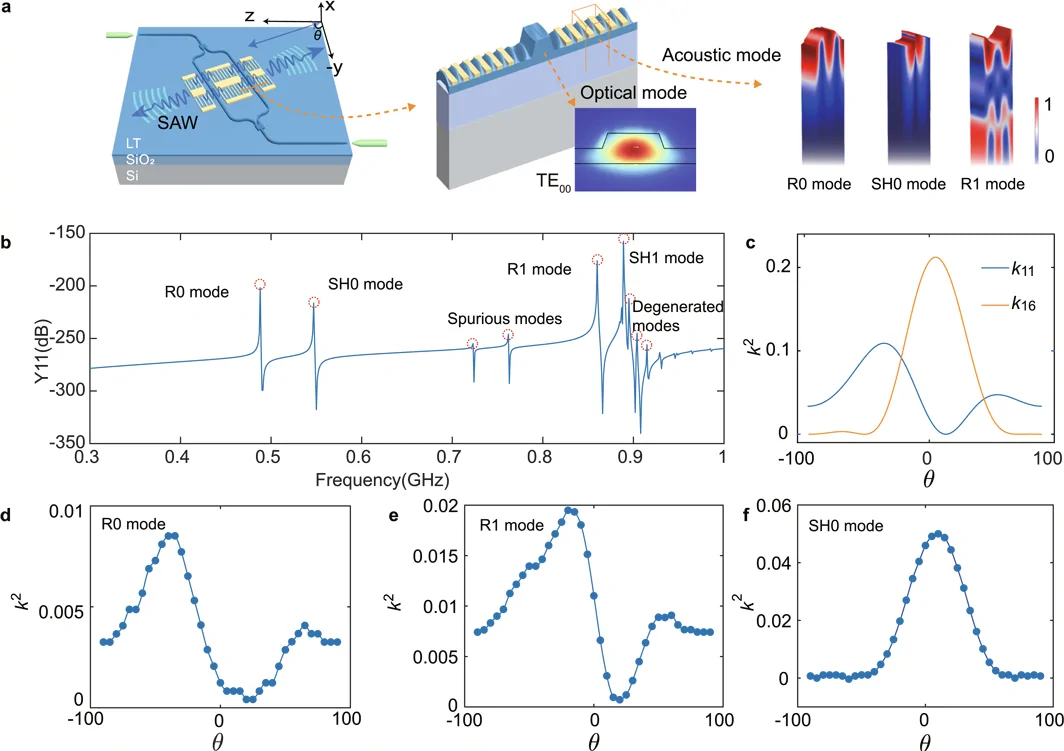
Design and simulation. (a) Schematic layouts of the AO modulators. Light is coupled to and from the chip through inverse tapers (green). The IDT (yellow) launches an SAW, and acoustic reflectors enhance the field in the cavity. Cross‐sectional optical and acoustic mode profiles are also shown. The MZI‐arm spacing provides the anti‐symmetric acoustic phase required for push–pull modulation. (b) Simulated $Y_{11}$ spectrum of the LTOI acoustic resonator, normalized to 1 S (0 dB). (c) Static $k^{2}$ of X‐cut LT; $k_{11}$ and $k_{16}$ denote the Rayleigh and shear‐horizontal contributions, respectively. (d–f) Simulated effective $k^{2}$ versus propagation angle for the R0, R1, and SH0 modes.

A finite‐element model containing the IDT was used to calculate the acoustic eigenfrequencies, mode profiles, and electromechanical coupling. The principal modes were identified as R0, SH0, and R1 from their displacement profiles [33, 34, 35]; their resonances are labeled in the simulated admittance spectrum in Figure 1b. The electromechanical coupling coefficient is defined as [36]

$$k^{2}\;=\;\frac{U_{I}^{2}}{U_{M}U_{E}}$$

where $U_{I}$, $U_{M}$, and $U_{E}$ are the mutual‐coupling, mechanical, and electrical energy terms, respectively. Taking −Y as the reference propagation direction (θ=0°), the static calculation gives maximum $k^{2}$ values of approximately 11% for the Rayleigh mode near −30° and 21% for the SH mode near 10° (Figure 1c) [37]. Simulations reproduce the angular trends for R0, R1, and SH0 (Figure 1d–f), with smaller values because part of the acoustic energy leaks into the buried oxide [28]. This anisotropy motivates the orientation‐dependent measurements below [38, 39, 40].

## 3 MZI‐Type AO Modulator and Its Anisotropy

Prior studies have enhanced AO coupling through suspended structures, high‐photoelastic materials, and optical microcavities [41, 42, 43]. Here, we focus on in‐plane crystal orientation and acoustic‐mode selection. The MZI was measured using the setup in Figure 2a. The electrode–waveguide spacing was optimized for the targeted acoustic modes (Supporting Information S1: Note S2). Lensed‐fiber coupling produced a fiber‐to‐fiber insertion loss of 12 dB, and the laser was set to the 50% transmission point at 1530 nm (Figure 2b).

**FIGURE 2:**
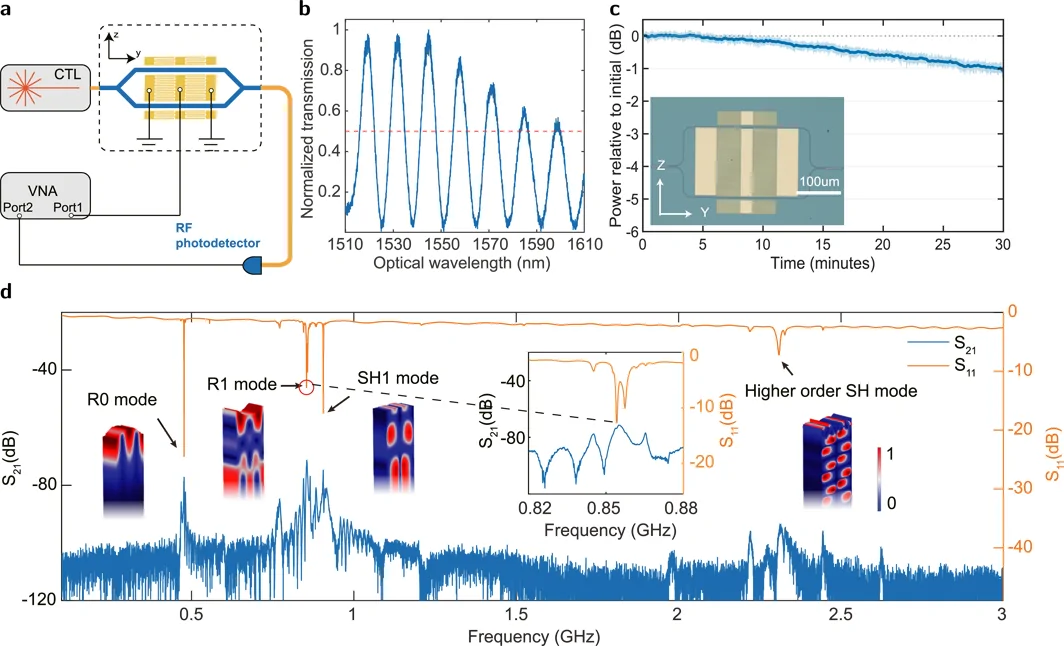
Characterization and output stability of the AO MZI. (a) Simplified experimental schematic. CTL, continuously tunable laser; PD, photodetector (22.5 V/W sensitivity); VNA, vector network analyzer. (b) Optical transmission of the AO MZI; the dashed line marks the selected 50% transmission point. (c) Thirty‐minute detected‐output record under a continuous 0.858‐GHz, 20‐dBm RF drive and 10‐dBm input optical power. The light‐blue trace is the raw record, the dark‐blue line is the 10‐s average, and the inset is a micrograph of the device. (d) Measured acoustic $S_{11}$ and acousto‐optic $S_{21}$ spectra.

To evaluate operating stability in the same MZI configuration, we recorded the detected output for 30 min under a continuous 0.858‐GHz, 20‐dBm RF drive and a 10‐dBm input optical power (Figure 2c). The 10‐s‐averaged output changed by approximately −0.99 dB; the full trace and measurement details are provided in Supporting Information S1: Note S11 and Figure S13.

The modulation efficiency was extracted from $S_{21}$. Port 1 of the VNA drives the IDT, and port 2 monitors the photoreceiver output. The measured $S_{11}$ and $S_{21}$ spectra (Figure 2d) contain resonances near 0.478, 0.858, 0.895, and 2.38 GHz, assigned to R0, R1, SH1, and a higher‐order SH mode, respectively. The SH0 mode is suppressed at θ=90°, consistent with the simulations in Supporting Information S1: Note S1. Fine splitting around the main resonances is attributed to the interaction of intrinsic modes with longitudinal cavity modes introduced by the acoustic reflectors (Supporting Information S1: Note S3).

Each resonance peak in the $S_{11}$ spectrum corresponds to a clear peak in the $S_{21}$ response, indicating AO modulation by multiple acoustic modes (Figure 2d). The R1 mode produces the strongest response because its overlap with the optical mode exceeds those of the R0 and SH0 modes, as detailed in Supporting Information S1: Note S3 and Figure S6. The half‐wave voltage $\left(V_{\pi }\right)$ of the MZI was extracted from the $S_{21}$ measurements using [41]

$$S_{21}\;=\;{\left(\frac{\pi R_{PD}I_{rec}}{V_{\pi }}\right)}^{2},$$

where $R_{PD}$ and $I_{rec}$ are the photoreceiver responsivity and detected optical power. At the 0.855‐GHz R1 resonance, $V_{\pi }=34.17$ V and $V_{\pi }L=0.68$ V⋅cm.

To investigate anisotropic AO modulation, we characterized the R0 mode in six devices oriented at −60°, −30°, 0°, 30°, 60°, and 90°. The $k^{2}$ values extracted from $S_{11}$ agree with the FEM results (Figure 3g). The maximum acoustic quality factor is $Q_{m}=8750$, the highest reported for a non‐suspended ferroelectric AO modulator [39]. The extracted $Q_{m}$, $k^{2}$, and $V_{\pi }L$ values are summarized in Table 1. At resonance, the modulation efficiency is governed by $Q_{m}$ and the AO coupling factor Γ, whereas $k^{2}$ primarily determines the impedance‐matching bandwidth [36, 41]. The corresponding figure of merit scales as $FOM_{AO}\;\propto \;Q_{m}\Gamma^{2}$ (Supporting Information S1: Note S4).

**FIGURE 3:**
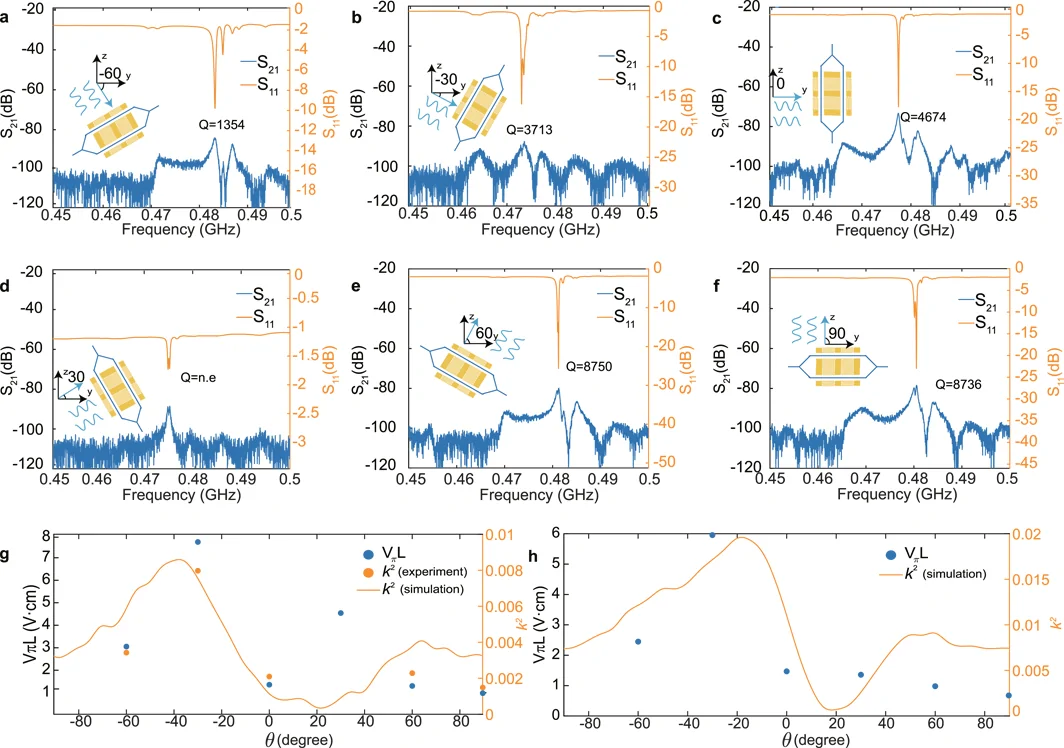
Orientation dependence of AO modulation in lithium tantalate. (a–f) Measured $S_{11}$ and $S_{21}$ spectra of the R0 mode at θ=−60°, −30°, 0°, 30°, 60°, and 90°, respectively. (g) Measured $V_{\pi }L$, experimental $k^{2}$, and simulated AO‐overlap trend for R0. (h) Measured $V_{\pi }L$ and simulated $k^{2}$ for R1. The notation “n.e.” indicates that $Q_{m}$ could not be reliably extracted because the R0 mode is suppressed at 30°.

**TABLE 1:** Measured orientation‐dependent parameters for the R0 mode. n.e., not evaluable because the mode is suppressed at 30°.

θ	$Q_{m}$	$k^{2}$ (exp.)	$V_{\pi }L$ (V⋅cm)
−60°	1354	∼0.005	3.053
−30°	3713	∼0.013	7.702
0°	4674	∼0.004	1.357
30°	n.e.	∼0	4.542
60°	8750	∼0.002	1.303
90°	8736	∼0.002	0.979

The data reveal a clear inverse correlation between $V_{\pi }L$ and $Q_{m}$: the highest‐$Q_{m}$ orientations (60° and 90°) deliver the lowest $V_{\pi }L$ values (1.303 and 0.979 V⋅cm). The −30° orientation, despite the largest $k^{2}$, exhibits the poorest $V_{\pi }L$ (7.702 V⋅cm), consistent with the well‐known $k^{2}$–$Q_{m}$ trade‐off in SAW resonators [36]. The R1 mode benefits from stronger refractive‐index modulation within the waveguide core, contributing to the record‐low $V_{\pi }L$ at 90° (see Supporting Information S1: Notes S3 and S4). A complete discussion of the orientation dependence, including FEM‐extracted Γ values and fabrication‐induced deviations from simulations, is provided in Supporting Information S1: Note S4.

## 4 Racetrack‐Type AO Modulator

Compared with MZI structures, racetrack modulators exhibit higher modulation efficiency owing to resonant enhancement of photon–phonon coupling in the optical cavity. The compact acoustic resonator is well suited to the racetrack cavity. The fabricated racetrack cavity achieved an optical quality factor $\left(Q_{L}\right)$ of $3.17\times 10^{5}$ for the TE‐polarized mode at a wavelength of 1552.7 nm (Supporting Information S1: Note S5). The strongest acoustic resonance occurs at 0.478 GHz, with $Q_{m}=3214$ (Figure 4b). At the optimized optical power, $S_{21}=-35.4$ dB, corresponding to $V_{\pi }=1.2$ V and $V_{\pi }L=0.024$ V⋅cm for the 200‐μm interaction length (Figure 4c).

**FIGURE 4:**
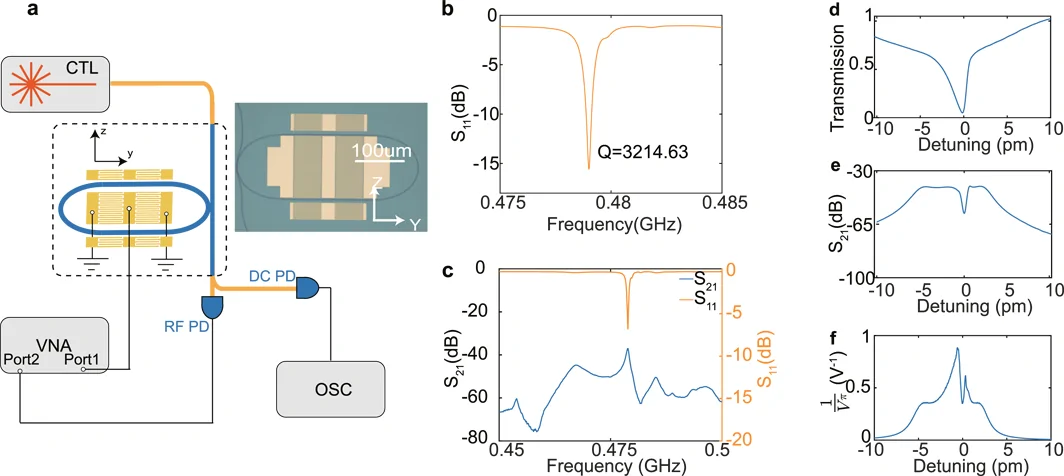
Characterization of the AO racetrack resonator. (a) Simplified experimental schematic. CTL, continuously tunable laser; PD, photodetector; VNA, vector network analyzer; OSC, oscilloscope. (b) Acoustic $S_{11}$ reflection spectrum. (c) Measured acoustic $S_{11}$ and acousto‐optic $S_{21}$ spectra. (d) Optical transmission, (e) $S_{21}$, and (f) $V_{\pi }$ versus cavity detuning.

In racetrack modulators, the modulation‐induced change in optical intensity is inherently linked to the resonance characteristics of the cavity transmission spectrum [43]. The modulation strength therefore depends on cavity detuning. To systematically probe this effect, we constructed a setup comprising a CTL, a VNA, a PD, and an OSC, allowing synchronous acquisition of optical transmission and $S_{21}$ (Figure 4a). The input wavelength was swept by the CTL while the VNA was fixed at 0.478 GHz. The response is largest on the blue‐detuned side (Figure 4d–f). With $\Delta =\omega_{\text{cavity}}-\omega_{\text{laser}}$, this condition has Δ<0, where Stokes scattering can provide negative optical damping and amplify the mechanical motion [11] (Supporting Information S1: Note S8). Under optimized blue‐detuned conditions, a minimum $V_{\pi }$ of 1.1 V is obtained, corresponding to a record‐low $V_{\pi }L$ of 22 mV⋅cm for the 200‐μm resonator.

## 5 Microwave‐Driven Optical Sideband Generation

The AO modulators developed in this work enable applications in microwave‐to‐optical conversion [44, 45], a function critical for low‐loss, long‐haul microwave‐signal transport and on‐chip photonic signal processing. To demonstrate this capability, we fabricated a racetrack AO modulator with 500‐nm‐wide electrodes for higher‐frequency operation (Supporting Information S1: Note S2). The optical racetrack has a loaded quality factor $Q_{L}=5.2\times 10^{5}$ at 1559 nm. At the 1.441‐GHz acoustic resonance, $S_{21}$ reaches −58.6 dB, giving $V_{\pi }=2.4$ V and $V_{\pi }L=0.048$ V⋅cm for the 200‐μm interaction length (Figure 5b).

**FIGURE 5:**
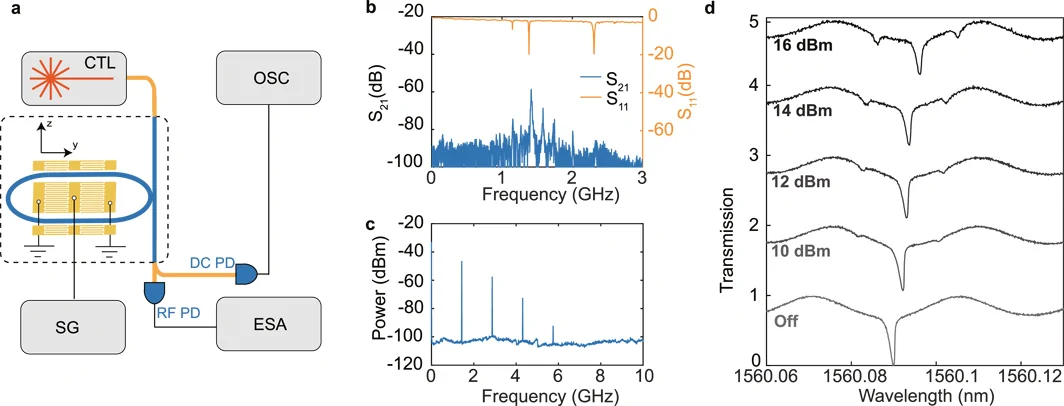
Demonstration of a microwave‐photonic link. (a) Simplified experimental schematic. CTL, continuously tunable laser; PD, photodetector; SG, signal generator; OSC, oscilloscope; ESA, spectrum analyzer. (b) Measured acoustic $S_{11}$ and acousto‐optic $S_{21}$ spectra. (c) Microwave spectrum of the photodetector output with a 16‐dBm RF drive applied to the acoustic resonator. The laser is blue‐detuned. (d) Optical transmission of the racetrack cavity versus RF power; the additional dips arise from AO modulation sidebands.

To evaluate the performance of our device in a microwave‐photonic link, we constructed a system comprising an electrical spectrum analyzer (ESA), a PD, a signal generator (SG), and an oscilloscope (OSC) (Figure 5a). As the RF drive increases to 16 dBm, additional transmission dips appear (Figure 5d) and are assigned to sidebands at $f_{\text{opt}}\pm nf_{a}$. The corresponding RF spectrum detected by the PD is displayed in Figure 5c. In the high‐Q racetrack resonator, these sidebands couple into the cavity resonance modes during the laser‐wavelength scan. The increasing depth and number of these sidebands with higher RF power directly demonstrate the high microwave‐to‐optical conversion efficiency of the non‐suspended LTOI platform.

## 6 Discussion and Outlook

The performance of the LTOI AO modulators is summarized in Table 2. Measurements across six crystal orientations show that the resonant modulation efficiency is primarily governed by the acoustic quality factor $Q_{m}$ and the acousto‐optic overlap Γ, whereas $k^{2}$ mainly affects the impedance‐matching bandwidth [36]. The non‐suspended MZI reaches $Q_{m}=8750$, and the measured $V_{\pi }L$ values are competitive with suspended LN, LN‐on‐sapphire, AlN, and $As_{2}S_{3}$‐based modulators [39, 41, 42, 43, 46, 48]. The use of multiple acoustic modes and an optical racetrack cavity further enables operation across several frequency bands and microwave‐driven optical sideband generation.

**TABLE 2:** Comparison with previously reported AO modulators.

Ref.	Platform	Suspended	Type	Frequency (GHz)	$Q_{m}$	$Q_{L}$	$V_{\pi }L$ (V⋅cm)
[46]	AlN on Si	n	FP	2.250	/	/	0.330
[39]	X‐cut LN on sapphire	n	MZI	0.700–0.800	600	/	0.940
[41]	X‐cut LN	y	MZI	3.330	3600	/	0.046
[41]	X‐cut LN	y	Racetrack	2.000	1562	$2.2\times 10^{6}$	0.0077
[47]	Si on X‐cut LNOI	n	MZI	0.496	/	/	0.496
[48]	$As_{2}S_{3}/SiO_{2}$/Y‐cut LN	n	MZI	0.11	/	/	0.94
[42]	$As_{2}S_{3}$ on X‐cut LN	n	MZI	0.84	202.81	/	0.03
[43]	$As_{2}S_{3}$ on X‐cut LN	n	Racetrack	0.84	/	$2.58\times 10^{5}$	0.009
This work	X‐cut LTOI	n	MZI	0.855	8750	/	0.680
This work	X‐cut LTOI	n	Racetrack	0.478	3214	$3.17\times 10^{5}$	0.022
This work	X‐cut LTOI	n	Racetrack	1.441	921	$5.2\times 10^{5}$	0.048

Material stability was evaluated using acoustic resonators with identical design parameters on LT and LN wafers over 20–50 °C. The extracted TCF demonstrates lower temperature‐induced resonance drift in LT (Supporting Information S1: Note S10 and Figure S12). LT has also been reported to exhibit weaker photorefractive effects than LN [49]; the 30‐min MZI measurement described above is consistent with sustained operation under simultaneous optical and RF excitation, with the full trace provided in Supporting Information S1: Note S11 and Figure S13.

Further development will focus on reducing device footprint and improving optical and acoustic confinement. Curved IDTs, partial acoustic isolation or phononic‐crystal boundaries, and compact Bragg cavities could shorten the interaction length, while wider waveguides and wafer‐scale DUV lithography with hard‐mask etching could improve $Q_{L}$ by reducing sidewall scattering [19]. Higher‐frequency operation may be obtained by redesigning the acoustic resonator or integrating LT with higher‐acoustic‐velocity materials [28]. These improvements could support optical‐frequency‐comb generation, on‐chip routing, and optical mode conversion [50, 51, 52].

## Author Contributions

**Weiran Zhou:** writing – original draft. **Chengli Wang:** writing – original draft. **Xuqiang Wang:** writing – original draft. **Bowen Chen:** writing – original draft. **Jiachen Cai:** writing – original draft. **Tianyao Yang:** writing – original draft. **Dongchen Sui:** writing – original draft. **Xinjian Ke:** writing – original draft. **Yang Chen:** writing – original draft. **Xudong Wang:** writing – original draft. **Ailun Yi:** writing – original draft. **Shibin Zhang:** writing – original draft. **Chengjie Zuo:** writing – original draft. **Xin Ou:** writing – original draft.

## Conflicts of Interest

The authors declare no conflicts of interest.

## Supporting information


Supporting Information S1

## Data Availability

The code and data underlying the results presented in this work are available at Zenodo (https://doi.org/10.5281/zenodo.22119638).
