# Supplementary material for: High‐Efficiency Acousto‐Optic Modulation on Non‐Suspended Thin‐Film Lithium Tantalate
Source: Nanophotonics. 2026 Aug 28;15(17):e70275. doi: 10.1002/nap2.70275 (PMC13672143; doi:10.1002/nap2.70275)
Supplement: Supplementary file 1 [file NAP2-15-e70275-s001.pdf]

Supplementary Information:
High-Efficiency Acousto-Optic Modulation on Non-Suspended Thin-Film Lithium Tantalate

Weiran Zhou,[1,2] Chengli Wang,[2,3,*] Xuqiang Wang,[2,3] Bowen Chen,[2,3] Jiachen Cai,[2,3] Tianyao Yang,[2,3] Dongchen Sui,[2,3] Xinjian Ke,[4] Yang Chen,[4] Xudong Wang,[2,3] Ailun Yi,[2,3] Shibin Zhang,[2,3] Chengjie Zuo,[1,†] and Xin Ou[1,2,3,4,‡]

[1]*School of Microelectronics, University of Science and Technology of China, Hefei, China*
[2]*State Key Laboratory of Materials for Integrated Circuits,*
Shanghai Institute of Microsystem and Information Technology,
Chinese Academy of Sciences, 865 Changning Road, Shanghai 200050, China.
[3]*Center of Materials Science and Optoelectronics Engineering,*
University of Chinese Academy of Sciences, Beijing 100049, China
[4]*Shanghai Novel Si Integration Technology, Shanghai 201815, China*

Supplementary Note S1. Electromechanical coupling calculation and angle-dependent SAW simulation

The electromechanical coupling coefficient can be expressed as follows [1]:

$$k_{i,j} = \left(\frac{e_{ij}}{\varepsilon_{ii} c_{jj}}\right)^2 \tag{S1}$$

where $i = 1, 2, 3...6$ denotes the x, y, z, yz, zx, xy direction in the biaxial crystal. Here, k_{ij} denotes the static electromechanical coupling coefficient when applying an electric field E_i and a stress field S_j. e_{ij}, ε_{ii} and c_{jj} are the components of the piezoelectric constant, dielectric constant, and elastic compliance.

The original form and values of tensor elements for e, ε and c in the crystalline principal axes have been well studied and are given in the references [2] as follows:

$$e = \begin{bmatrix} 0 & 0 & 0 & 0 & e_{15} & -2e_{22} \\ -e_{22} & e_{22} & 0 & e_{15} & 0 & 0 \\ e_{31} & e_{31} & e_{33} & 0 & 0 & 0 \end{bmatrix},$$

$$c = \begin{bmatrix} c_{11} & c_{12} & c_{13} & c_{14} & 0 & 0 \\ c_{12} & c_{11} & c_{13} & c_{14} & 0 & 0 \\ c_{13} & c_{13} & c_{33} & 0 & 0 & 0 \\ c_{14} & c_{14} & 0 & c_{44} & 0 & 0 \\ 0 & 0 & 0 & 0 & c_{44} & c_{14} \\ 0 & 0 & 0 & 0 & c_{14} & \frac{(c_{11}-c_{12})}{2} \end{bmatrix},$$

$$\varepsilon = \begin{bmatrix} \varepsilon_{11} & 0 & 0 \\ 0 & \varepsilon_{11} & 0 \\ 0 & 0 & \varepsilon_{13} \end{bmatrix}. \tag{S2}$$

In the transformed coordinate system x'–y'–z', obtained by successive rotations of the original frame—first by an angle θ about the z axis, then by 90° about the new x axis, and finally by 90° about the resulting z axis—the tensor components are transformed via the transformation matrices **a** and **M** as follows:

$$\alpha = \begin{bmatrix} \cos\theta & \sin\theta & 0 \\ -\sin\theta & \cos\theta & 0 \\ 0 & 0 & 1 \end{bmatrix} \times \begin{bmatrix} 1 & 0 & 0 \\ 0 & 0 & 1 \\ 0 & -1 & 0 \end{bmatrix} \times \begin{bmatrix} 0 & 1 & 0 \\ -1 & 0 & 0 \\ 0 & 0 & 1 \end{bmatrix} = \begin{bmatrix} 0 & \cos\theta & \sin\theta \\ 0 & -\sin\theta & \cos\theta \\ 1 & 0 & 0 \end{bmatrix}$$

* wangcl@mail.sim.ac.cn
† czuo@ustc.edu.cn
‡ ouxin@mail.sim.ac.cn

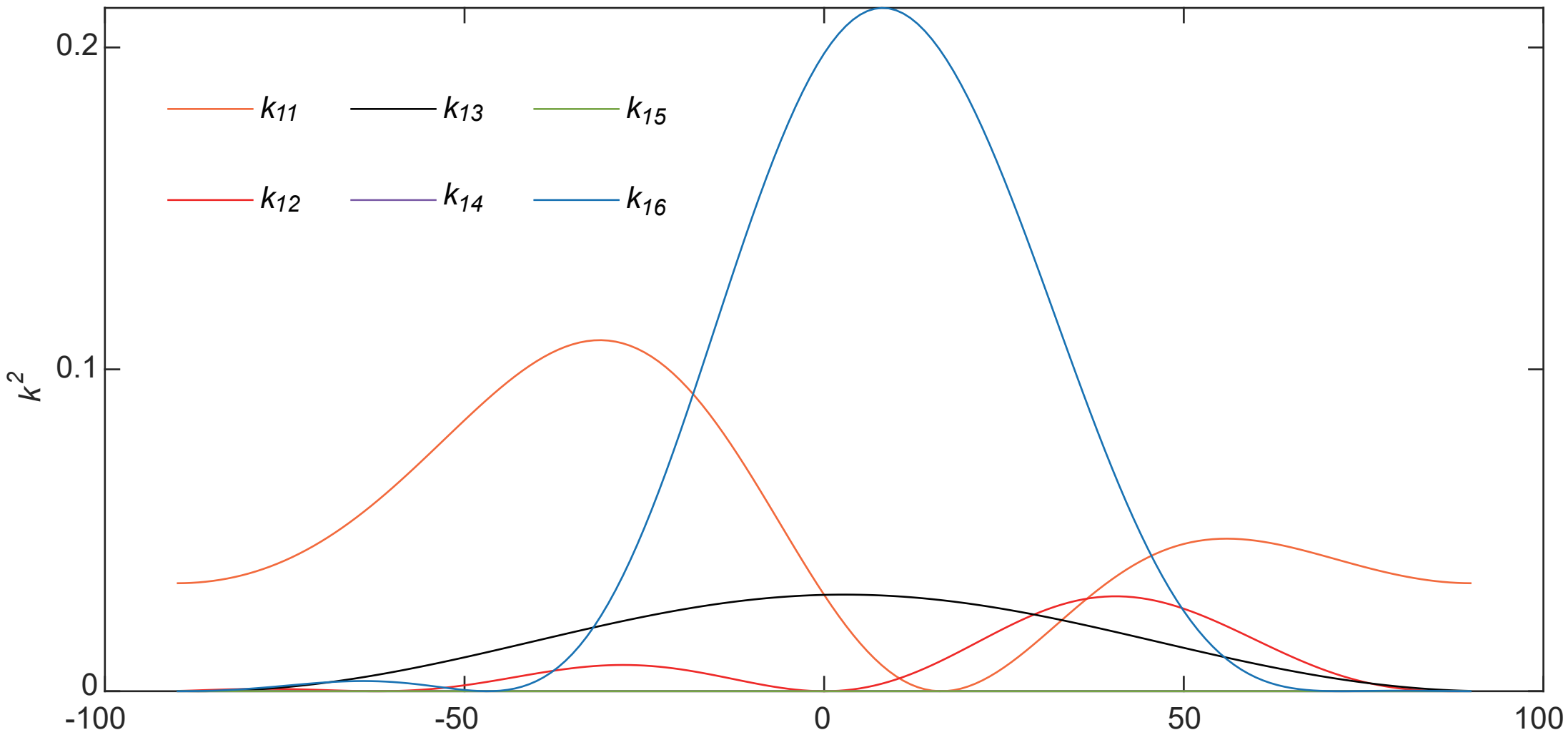

FIG. S1. Calculation result of electromechanical coupling coefficient. $k_{11}, k_{12}, k_{13}, k_{14}, k_{15}, k_{16}$ refer to components of electromechanical coupling coefficient when applying electric field along x axis of device plane.

$$M = \begin{bmatrix} 0 & \cos^2\theta & \sin^2\theta & 2\cos\theta\sin\theta & 0 & 0 \\ 0 & \sin^2\theta & \cos^2\theta & -2\sin\theta\cos\theta & 0 & 0 \\ 1 & 0 & 0 & 0 & 0 & 0 \\ 0 & 0 & 0 & 0 & \cos\theta & -\sin\theta \\ 0 & 0 & 0 & 0 & \sin\theta & \cos\theta \\ 0 & -\cos\theta\sin\theta & \cos\theta\sin\theta & \cos^2\theta-\sin^2\theta & 0 & 0 \end{bmatrix}$$

$$e' = aeM^T; \quad c' = McM^T; \quad \varepsilon' = a\varepsilon a^T. \tag{S3}$$

Substituting Eq. S2 and Eq. S3 into Eq. S1 yields the electromechanical coupling coefficient of lithium tantalate, as illustrated in Fig. S1. The resulting curve indicates that the excitation of surface acoustic waves in lithium tantalate is predominantly influenced by k_{11} and k_{16}, which correspond to the electromechanical coupling coefficients of the Rayleigh and shear modes, respectively.

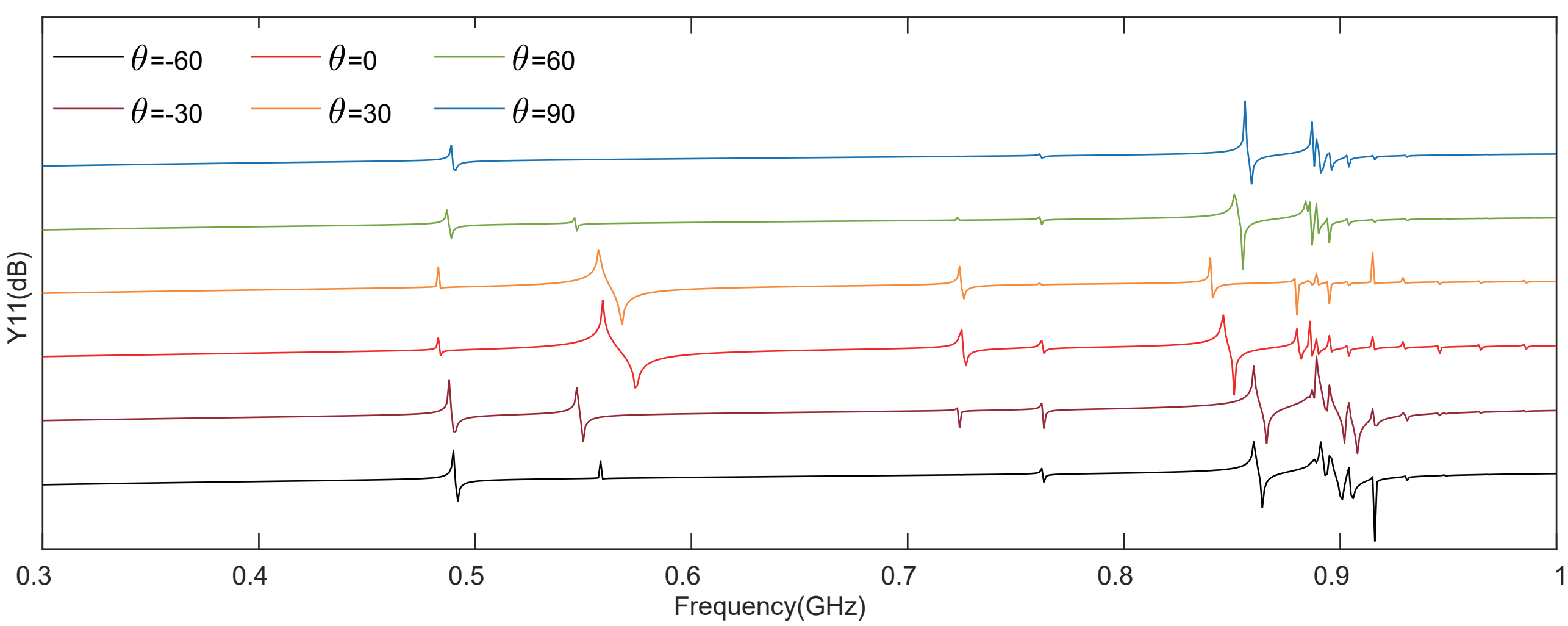

FIG. S2. FEM simulation of admittance curve of acoustic resonator with different propagation angle on LTOI.

Specifically, the 3D FEM simulations were conducted using a unit cell representing a single period of the IDT electrode structure to match the target wavelength. Periodic boundary conditions (PBC) were applied along the propagation direction to simulate

an infinite periodic structure. This method avoids the massive computational resources required to mesh the entire resonant cavity (comprising hundreds of IDT finger pairs) while enabling the precise extraction of phase velocities and cross-sectional mode shapes used for calculating the acousto-optic overlap integral. Utilizing this model, FEM simulations were performed to investigate the influence of propagation angle on the excitation efficiency of surface acoustic wave (SAW) modes. The results are presented in Fig. S2. As evidenced by the obtained curves, the R0 mode is most efficiently excited at a propagation angle of $\theta = -30°$. Conversely, a distinct suppression of the R0 mode is observed at $\theta = 30°$. This angular selectivity is mathematically attributed to the transformation of the piezoelectric tensor components in the rotated coordinate system: at $\theta = 30°$, the effective piezoelectric coefficient e'_{31} in the rotated frame approaches zero, resulting in vanishing electromechanical coupling for the R0 mode. This explains the absence of a measurable resonance and the "n.e." (not evaluable) designation for Q_m at this orientation in the main text. In contrast, the SH0 mode exhibits optimal excitation efficiency at $\theta = 0°$. The R1 mode demonstrates high excitation efficiency across a range of angles, with the notable exception of $\theta = 30°$.

Supplementary Note S2. AOM geometry optimization and device fabrication

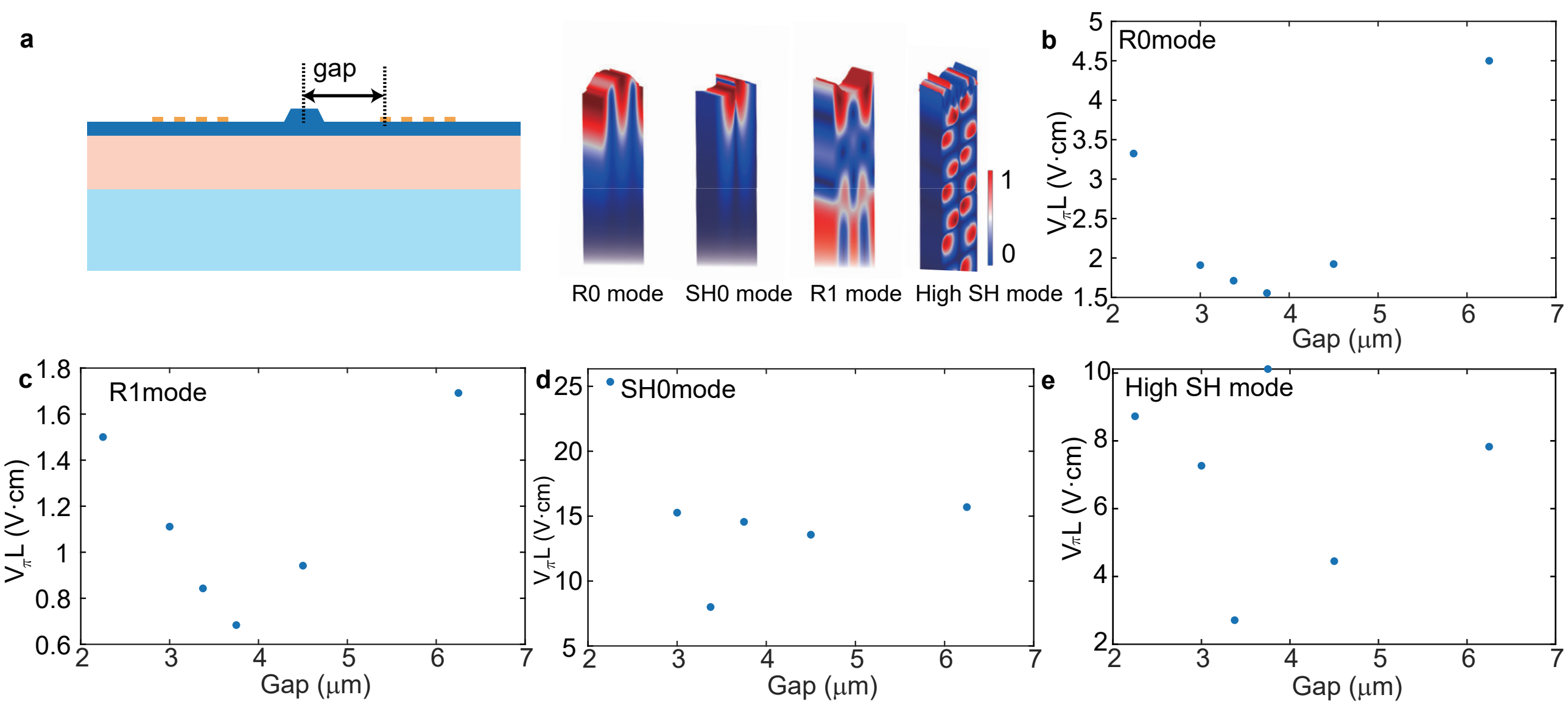

FIG. S3. **Optimization of waveguide-electrode spacing.** (a) Experimental setup for AOMZI characterization. (b) Modulation efficiency of R0 mode versus spacing. (c) Modulation efficiency of R1 mode versus spacing. (d) Modulation efficiency of SH0 mode versus spacing. (e) Modulation efficiency of higher-order SH modes versus spacing.

To further optimize the performance of the acousto-optic modulator, the spacing between the electrode and the waveguide was systematically varied to maximize the acousto-optic coupling efficiency. This optimization is essential to achieve the push-pull condition described in the main text. By adjusting the gap, we ensure that the acoustic phase difference between the two waveguide arms approximates π (180°), thereby maximizing the differential phase shift. A series of Mach–Zehnder interferometer-type acousto-optic modulators (AOMZIs) with acoustic wave propagation along the Z-axis of lithium tantalate were fabricated and characterized using the same experimental setup as described in the main text Fig. S3(a). The modulation efficiency of each AOMZI was measured across the frequency ranges corresponding to different acoustic modes while varying the electrode-waveguide spacing. The R1 mode, which exhibited the strongest acousto-optic coupling, achieved optimal modulation efficiency at a spacing of 3.375 μm, as summarized in Fig. S3(c). A similar trend was observed for both the R0 and SH0 modes Fig. S3(b,d), which can be attributed to their nearly identical acoustic wavelengths and mode profiles along the X-axis of the device. To further examine this dependence, higher-order shear-horizontal (SH) modes were also characterized Fig. S3(e), revealing a similar behavior due to their similar mode extent and energy distribution in the X axis. These results demonstrate that modulation efficiency can be substantially enhanced through precise optimization of the electrode-waveguide spacing.

Before device fabrication, the LTOI stack was prepared at the Novel Si integration technology(NSIT). The LTOI wafer was fabricated by the smart-cut technique. The fabrication process for the acousto-optic modulators (AOMs) is illustrated in Fig. S4(a). Using this process, a racetrack (RT) AOM with an acoustic wavelength of 6 μm was fabricated. Figure S4(b) shows an optical micrograph of the acousto-optic modulator, while Fig. S4(c) provides a detailed view of the IDT electrodes. Specifically, the IDT consists of 30 finger pairs with a uniform period and finger width throughout. No apodization or chirping was applied to the inner or outer electrodes. This uniformity ensures that the excited acoustic wavefront maintains a consistent phase relation, allowing the high-Q Fabry-Perot cavity formed by the external reflectors to effectively confine the acoustic energy and dominate

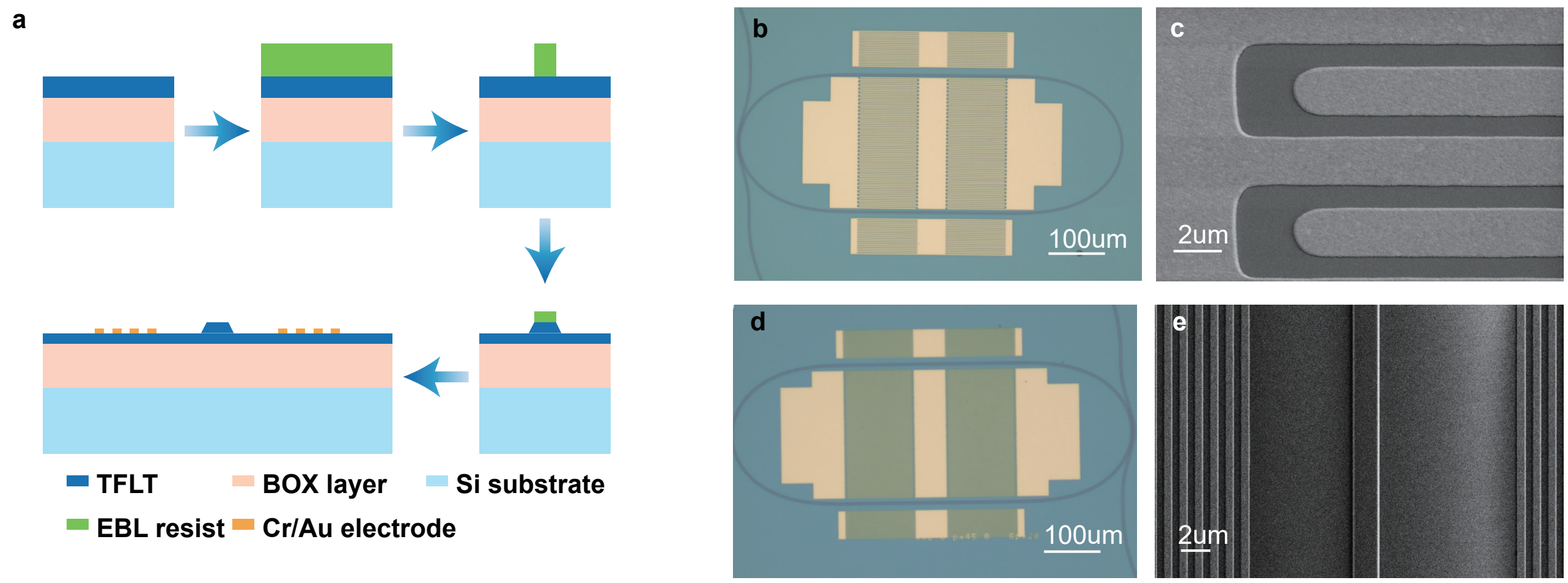

FIG. S4. **Fabrication and characterization of AOM.** (a) Schematic of the fabrication process of AOM. (b) Partial microscope image of the RT AOM at low frequency. (c) Partial SEM image of RT AOM at low frequency. (d) Partial microscope image of the RT AOM at high frequency. (e) Partial SEM image of RT AOM at high frequency.

the resonant response. For the microwave-driven optical sideband experiment discussed in the main text, an additional RT AOM with a reduced acoustic wavelength of 2 µm was fabricated following the same process flow. To achieve the finer IDT electrode features required at this scale, the maskless laser direct writing (MLA) alignment process described above was replaced with electron-beam lithography (EBL) alignment. A higher-resolution electron-beam resist, ZEP520A, was employed to accurately pattern the nanoscale electrode structures. Fig. S4(d) presents an overall optical micrograph of this device, and Fig. S4(e) shows a scanning electron microscopy (SEM) image detailing the IDT and optical waveguide structures. The visual variation between the reflectors of the MZI and racetrack modulators arises from a layout optimization for fabrication stability. While the early-batch MZI devices featured isolated grating lines, the subsequent racetrack resonators incorporated stabilizing bar electrodes at the grating ends. This modification was implemented to prevent electrode detachment during the metal lift-off process, ensuring higher structural integrity without altering the underlying acoustic design.

Supplementary Note S3. Acoustic-cavity mode structure and acousto-optic overlap

To elucidate the physical origin of the fine spectral structures ("degenerated modes") observed in the RF measurements and their dependence on propagation angle, we performed finite element method (FEM) simulations based on the fabricated device geometry. The simulation model incorporates **30 pairs of IDT electrodes and 10 pairs of reflector grating electrodes**.

1. Origin of Spectral Splitting: As shown in Fig. S5(a), the simulated admittance (Y_{11}) curve for the device *without* the grating structure already exhibits two distinct resonance peaks. The corresponding displacement profiles indicate that:

- The peak at 0.852 GHz corresponds to the main R_1 mode (Fig. S5(c)).
- The peak at 0.889 GHz corresponds to a secondary intrinsic mode arising from spatial variations in the vibration distribution (Fig. S5(e)).

Upon introducing the grating electrodes, as shown in Fig. S5(b), the spectral response evolves. A new resonance peak emerges at 0.865 GHz, located exactly between the two intrinsic modes. The mode profile of this specific peak, presented in Fig. S5(d), confirms that it is a **longitudinal cavity mode** confined effectively by the acoustic reflectors. Thus, the experimentally observed splitting is a superposition of intrinsic spatial degeneracy and the enhanced cavity mode.

2. Angle Dependence of Visibility: The experimental observation that these extra resonances are prominent only at specific angles (as noted in Fig. 3) is attributed to the strong anisotropy of X-cut $LiTaO_3$. The high-Q cavity mode (Fig. S6(a)) requires efficient acoustic reflection and strong electromechanical transduction to be observed. At optimal propagation angles (e.g., near $\theta = -30°$ for the Rayleigh mode), the effective electromechanical coupling coefficient (k^2_{eff}) is maximized, allowing this discrete longitudinal mode to be strongly excited and spectrally resolved. Conversely, at off-axis angles where coupling is weak or leakage is high, the cavity mode is not efficiently driven, causing the fine spectral splitting to broaden or disappear from the measured spectrum.

We calculated the acousto-optic overlap integral as a format of change of refractive index for the different acoustic modes (R0, R1, SH0) with the fundamental TE optical mode. The overlap integral is defined as:

$$\Delta n = \Delta n_{pe} + \Delta n_{eo} + \Delta n_{mb} \quad \text{(S4)}$$

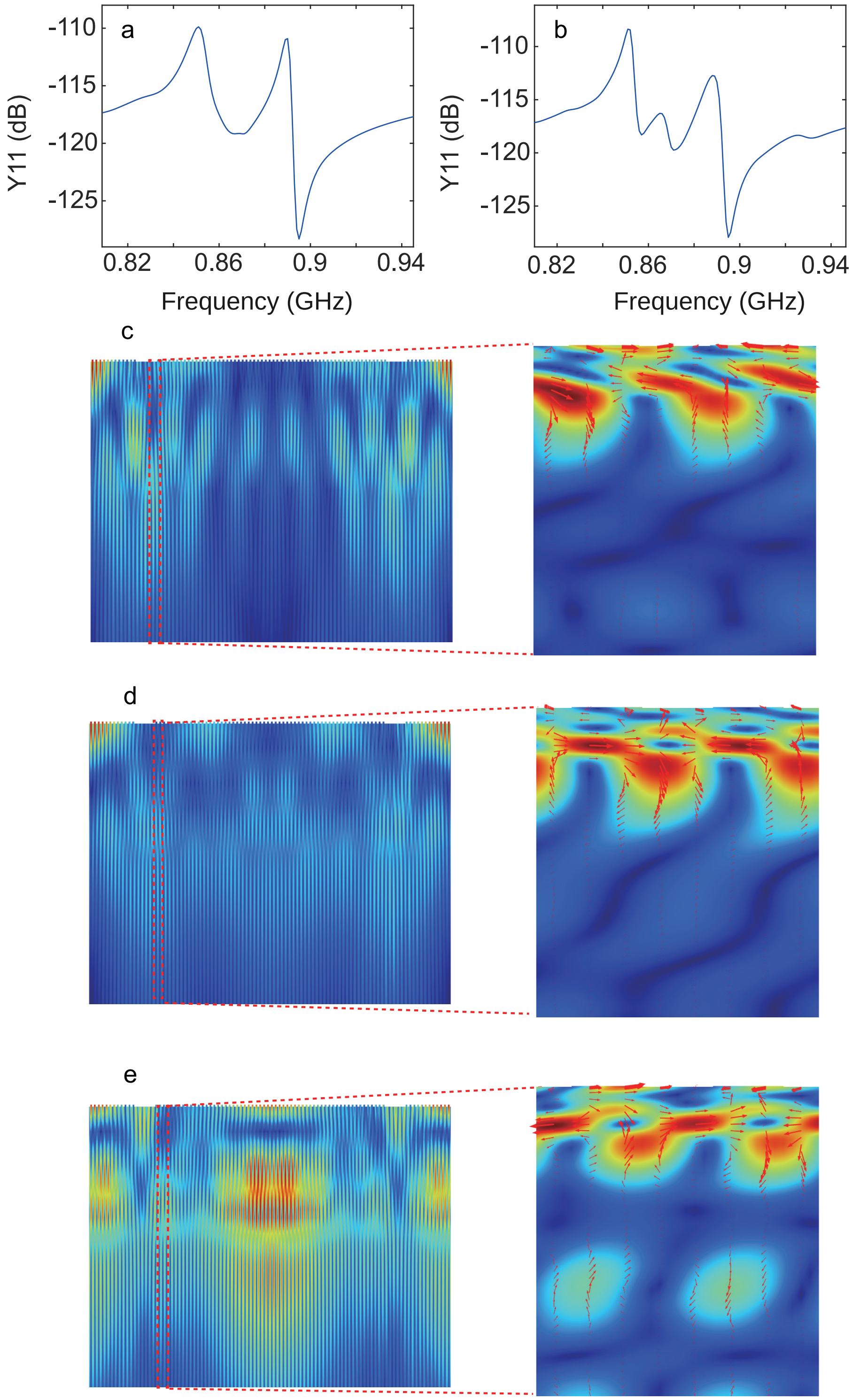

FIG. S5. **FEM simulation of the acoustic cavity effect.** The model incorporates 30 pairs of IDT electrodes and 10 pairs of reflector grating electrodes. (a) Simulated admittance (Y_{11}) curve *without* acoustic reflectors, showing two intrinsic modes. (b) Simulated Y_{11} curve *with* acoustic reflectors, showing the appearance of a new cavity mode between the intrinsic ones. (c) Mode profile of the main R_1 resonance at 0.852 GHz (Intrinsic). (d) Mode profile of the reflector-induced cavity mode at 0.865 GHz (New). (e) Mode profile of the intrinsic spatially degenerate mode at 0.889 GHz (Intrinsic).

Where Δn_{pe}, Δn_{eo}, Δn_{mb} refer to refractive index change caused by photo-elastic effect, electro-optic effect (electric field comes from acoustic wave through piezoelectric effect) and moving boundary effect. The calculation of these effects can be done by:

$$\Delta n_{pe,eo} = \frac{\epsilon_0 n^5}{2} \frac{\int dr (E_x^*, E_y^*, E_z^*) \begin{pmatrix} dB_1 & dB_6 & dB_5 \\ dB_6 & dB_2 & dB_4 \\ dB_5 & dB_4 & dB_3 \end{pmatrix} \begin{pmatrix} E_x \\ E_y \\ E_z \end{pmatrix}}{\int \vec{E}^* \epsilon \vec{E} dr} \tag{S5}$$

$$\Delta n_{mb} = -\frac{n}{2} \frac{\oint (\vec{R} \cdot \vec{n}) \left(\Delta\epsilon (\vec{E}^* \times \vec{n} \cdot \vec{E} \times \vec{n}) - \Delta\epsilon^{-1} (\vec{D}^* \cdot \vec{n})(\vec{D} \cdot \vec{n}) \right) dS}{\int \vec{E}^* \epsilon \vec{E} dr} \tag{S6}$$

Where $\vec{E}$ denotes that electric field of optic wave. R refers to displacement field. $\vec{D}$ is the electric displacement field of optical mode. To further numerically calculate the index change, the coefficient can be written as below:

$$\begin{pmatrix} dB_{1_{pe}} \\ dB_{2_{pe}} \\ dB_{3_{pe}} \\ dB_{4_{pe}} \\ dB_{5_{pe}} \\ dB_{6_{pe}} \end{pmatrix} = \begin{pmatrix} p_{33} & p_{31} & p_{31} & 0 & 0 & 0 \\ p_{13} & p_{11} & p_{12} & 0 & p_{14} & 0 \\ p_{13} & p_{12} & p_{11} & 0 & -p_{14} & 0 \\ 0 & 0 & 0 & p_{66} & 0 & p_{14} \\ 0 & p_{41} & -p_{41} & 0 & p_{44} & 0 \\ 0 & 0 & 0 & p_{41} & 0 & p_{66} \end{pmatrix} \begin{pmatrix} S_1 \\ S_2 \\ S_3 \\ S_4 \\ S_5 \\ S_6 \end{pmatrix} \tag{S7}$$

$$\begin{pmatrix} dB_{1_{eo}} \\ dB_{2_{eo}} \\ dB_{3_{eo}} \\ dB_{4_{eo}} \\ dB_{5_{eo}} \\ dB_{6_{eo}} \end{pmatrix} = \begin{pmatrix} r_{33} & 0 & 0 \\ r_{13} & 0 & -r_{22} \\ r_{13} & 0 & -r_{22} \\ 0 & -r_{22} & 0 \\ 0 & 0 & r_{51} \\ 0 & -r_{51} & 0 \end{pmatrix} \begin{pmatrix} \varepsilon_x \\ \varepsilon_y \\ \varepsilon_z \end{pmatrix} \tag{S8}$$

where p_{ij}, r_{ij} denote photoelastic and electro-optic coefficient. And $\vec{\varepsilon}$ refers to electric field induced by piezoelectric effect from acoustic wave.

For the quantitative calculation of the refractive index change, the simulation was performed with a microwave input power of 0.001 W. The resonant frequencies for these calculations were identified based on the admittance curves (Y_{11}) shown in Fig. S6(a).

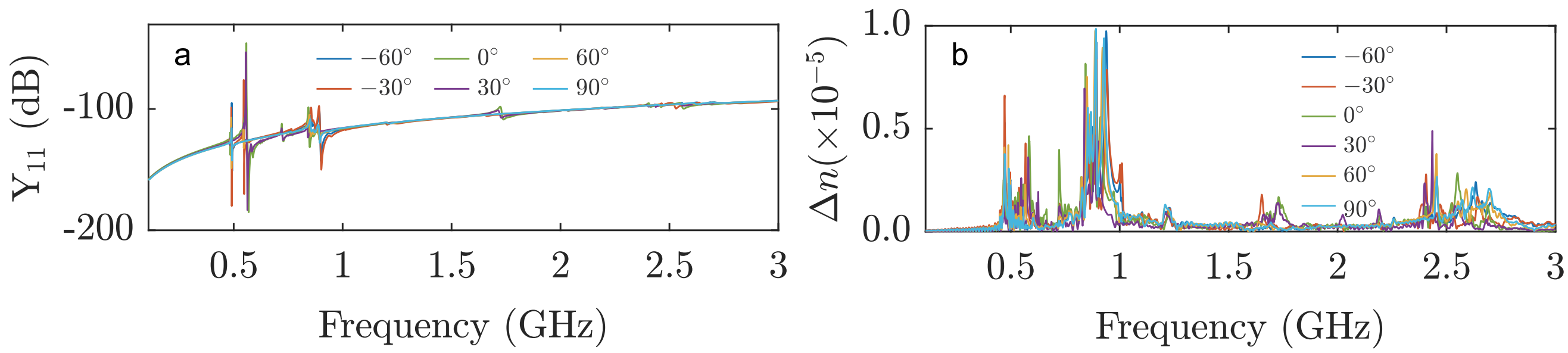

FIG. S6. **Quantitative analysis of acousto-optic coupling.** (a) Simulated Y_{11} admittance curves of the whole AOM structure with different in-plane propagation angles. (b) Calculated refractive index change (Δn) as a function of frequency, based on a microwave input power of 0.001 W. The peaks in (b) correspond to the acoustic resonant modes identified in (a). The simulation results indicate that at a propagation angle of $\theta = 90°$, the refractive index modulation reaches its maximum at 0.887 GHz, corresponding to the R1 mode.

Our calculations, as illustrated in Fig. S6, reveal that at a propagation angle of $\theta = 90°$, the maximum refractive index change occurs at a frequency of 0.887 GHz, which corresponds to the R1 mode. This overlap factor is significantly higher than that of the R0 and SH0 modes. This quantitative analysis confirms that the R1 mode profile has the highest spatial overlap with the optical mode within the waveguide core, leading to the higher measured modulation efficiency of the R1 mode.

Importantly, these calculations also reveal that the AO overlap integral Γ (quantified by Δn) is NOT maximized at $\theta = -30°$ where k^2 peaks. Instead, the optimal AO overlap occurs at $\theta = 90°$ for the R1 mode. This angular dependence of the overlap integral arises from the rotation of the acoustic displacement field relative to the optical waveguide axis—at 90°, the acoustic strain components that most effectively modulate the refractive index through the photoelastic and electro-optic tensors are preferentially aligned with the optical mode polarization. This finding underscores a key design principle: maximizing k^2 alone does not guarantee optimal AO modulation, because the spatial distribution of the acoustic field must also optimally overlap with the optical mode.

Supplementary Note S4. Orientation-dependent modulation and figure-of-merit analysis

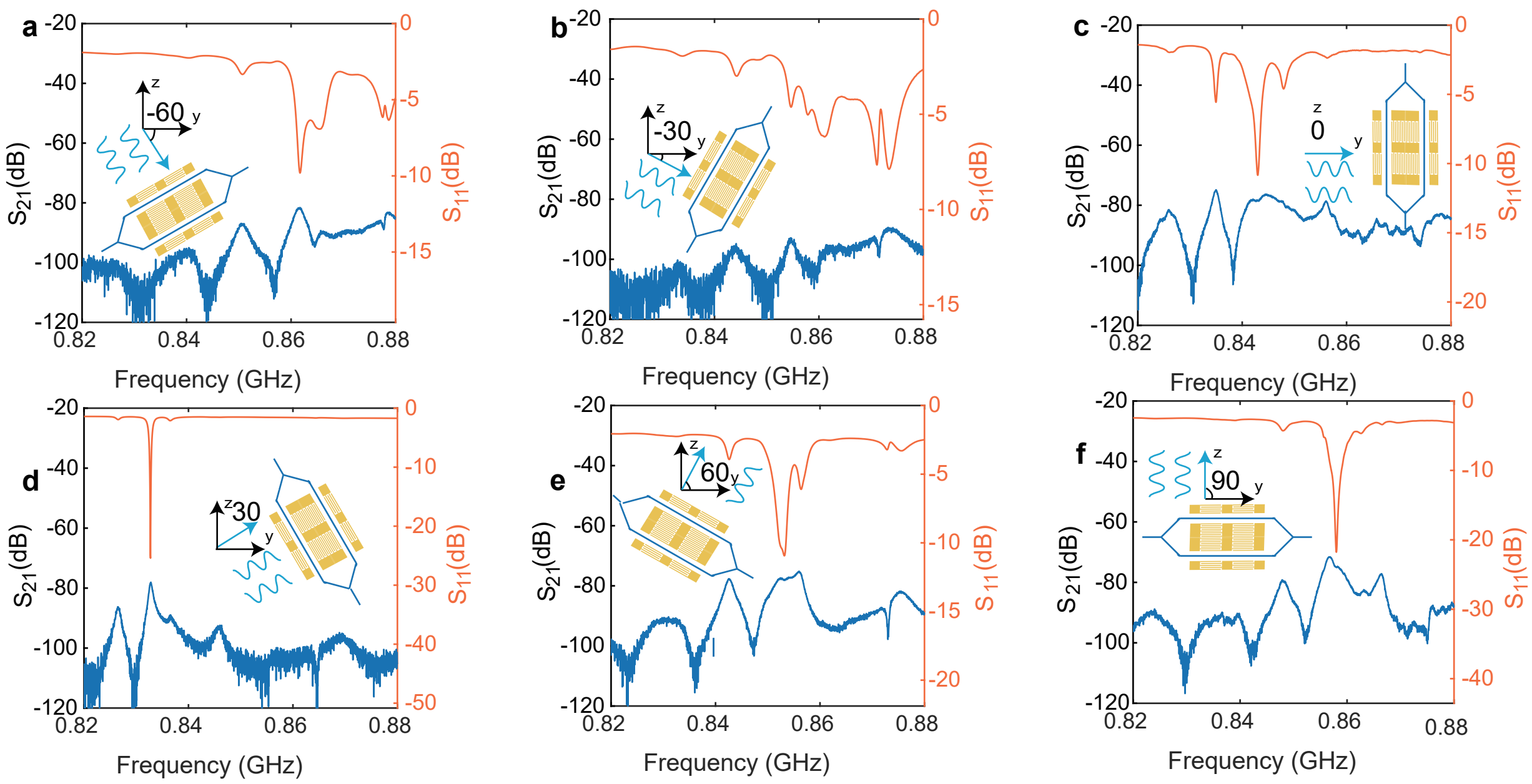

FIG. S7. **Characterization of the anisotropy of AO modulation in Lithium Tantalate.** (a) Measured S_{11} and S_{21} spectrum of R0 mode when θ = -60°, the optical power detected by the photoreceiver is 0.16 mW. (b) Measured S_{11} and S_{21} spectrum of R0 mode when θ = -30°, the optical power detected by the photoreceiver is 0.12mW. (c) Measured S_{11} and S_{21} spectrum of R0 mode when θ = 0°, the optical power detected by the photoreceiver is 0.2 mW. (d) Measured S_{11} and S_{21} spectrum when θ = 30°, The optical power detected by the photoreceiver is 0.1 mW. (e) Measured S_{11} and S_{21} spectrum when θ = 60°, The optical power detected by the photoreceiver is 0.11 mW. (f) Measured S_{11} and S_{21} spectrum when θ = 90°, The optical power detected by the photoreceiver is 0.1 mW.

Based on the standard extraction method of $V_\pi L$ for MZI AO modulators, the values of R0 mode for devices oriented at –60 °, –30°, 0°, 30°, 60° and 90° were determined to be 2.649, 5.954, 1.7865, 1.358, 1.0385, and 0.68 V · cm.

Note that unlike the R0 mode, the effective k^2 values for the R1 mode presented in Fig. 3(h) of the main text are derived from FEM simulations. As discussed in the main text, the experimental extraction of k^2 for R1 was precluded by the presence of dense longitudinal modes within the high-Q acoustic cavity, which obscured the distinct series and parallel resonance frequencies required for standard mBVD extraction.

This supplementary note provides a rigorous framework for understanding the orientation-dependent AO modulation efficiency presented in the main text, grounded in established acousto-optic and SAW resonator theory[3, 4]. We clarify the distinct roles of the three key parameters: the electromechanical coupling coefficient (k^2), the acoustic quality factor (Q_m), and the acousto-optic coupling factor (Γ).

1. **Electromechanical coupling coefficient (k^2):** This parameter quantifies the piezoelectric transduction strength and is defined as the ratio of mutual energy to the geometric mean of the electrical and mechanical stored energies[1, 3]. In SAW resonators, k^2 determines the spacing between the series (f_s) and parallel (f_p) resonance frequencies, which governs the electrical impedance matching bandwidth to the 50 Ω source[3]. Consequently, k^2 primarily determines the *modulation bandwidth* of the AO device—i.e., the frequency range over which efficient electrical-to-acoustic transduction can be maintained—rather than the steady-state modulation efficiency at resonance. A higher k^2 enables broader bandwidth operation but does not directly improve $V_\pi L$.

2. **Acoustic quality factor (Q_m):** This parameter characterizes the resonant enhancement of the acoustic energy density within the acoustic Fabry-Pérot cavity formed by the grating reflectors[4]. Under resonant drive, the stored acoustic energy is amplified by a factor of Q_m relative to the non-resonant case[3]:

$$U_\mathrm{acoustic} \propto Q_\mathrm{m} \cdot P_\mathrm{in} \tag{S9}$$

where P_in is the RF power delivered to the acoustic mode at the impedance-matched resonance.

3. **Acousto-optic coupling factor (Γ):** This parameter quantifies the spatial overlap between the acoustic strain field and the guided optical mode. The refractive index change Δn induced by the acoustic wave, mediated by the photoelastic, electro-optic, and moving-boundary effects[4], scales as:

$$\Delta n \propto \Gamma \cdot \sqrt{U_\mathrm{acoustic}} \propto \Gamma \cdot \sqrt{Q_\mathrm{m}} \tag{S10}$$

The normalized Γ values for the R0 mode at each orientation are calculated from FEM simulations, as detailed in Supplementary Note S3.

Since the phase shift in an MZI AO modulator is proportional to Δn, and V_π is the voltage required to induce a π phase shift, we obtain[4]:

$$\mathrm{V}_\pi \mathrm{L} \propto \frac{1}{(\Delta n)^2} \propto \frac{1}{\Gamma^2 \cdot Q_\mathrm{m}} \tag{S11}$$

Thus, the acousto-optic figure of merit is:

$$\mathrm{FOM}_\mathrm{AO} = Q_\mathrm{m} \cdot \Gamma^2 \tag{S12}$$

$$\boxed{\mathrm{V}_\pi \mathrm{L} \propto \frac{1}{Q_\mathrm{m} \cdot \Gamma^2}} \tag{S13}$$

We emphasize that k^2 does not appear in the final expression for $\mathrm{V}_\pi\mathrm{L}$, because at the impedance-matched resonance condition, the delivered RF power is independent of k^2. The role of k^2 is to determine the *bandwidth* over which this impedance matching can be maintained, not the *efficiency* at resonance.

Application to Experimental Data:

Table S1 summarizes the complete dataset: the measured Q_m, simulated coupling factor Γ (normalized to the maximum value), measured k^2, and measured $\mathrm{V}_\pi\mathrm{L}$ for all six orientations.

TABLE S1. Comprehensive analysis of orientation-dependent AO modulation parameters for the R0 mode. Γ (sim.) is the simulated coupling strength normalized to the maximum value (0.66 at −30°). k^2 is provided for completeness but primarily determines modulation bandwidth, not $\mathrm{V}_\pi\mathrm{L}$. n.e. = not evaluable.

θ	Q_m	Γ (sim.)	k^2 (exp.)	$Q_\mathrm{m} \cdot \Gamma^2$	$\mathrm{V}_\pi\mathrm{L}$ (V·cm)
−60°	1354	0.52	∼ 0.005	366	3.053
−30°	3713	0.66	∼ 0.013	1617	7.702
0°	4674	0.22	∼ 0.004	226	1.357
30°	n.e.	0.10	∼ 0	—	4.542
60°	8750	0.42	∼ 0.002	1544	1.303
90°	8736	0.37	∼ 0.002	1196	0.979

Discussion of Key Observations:

1. **The simulated coupling factor Γ does not predict $\mathrm{V}_\pi\mathrm{L}$.** The highest simulated Γ (0.66) occurs at −30°, yet this orientation yields the poorest $\mathrm{V}_\pi\mathrm{L}$ (7.702 V·cm). Conversely, 90° with a moderate Γ (0.37) achieves the best $\mathrm{V}_\pi\mathrm{L}$ (0.979 V·cm). The combined FOM $Q_\mathrm{m} \cdot \Gamma^2$ also fails to correctly rank the orientations—−30° has the highest FOM (1617) but the worst performance. This discrepancy indicates that the simulated Γ values, while useful for qualitative mode analysis, do not fully capture the performance of fabricated devices. The likely cause is fabrication-induced variations in the waveguide geometry, electrode positioning, and the precise acoustic mode profiles, which introduce deviations between the idealized simulation and the actual device overlap integral.

2. $V_\pi L$ **correlates strongly with Q_m.** Excluding the suppressed 30° case, the experimental $V_\pi L$ exhibits a clear monotonic trend with Q_m: orientations with higher Q_m consistently achieve lower $V_\pi L$ (better modulation efficiency). The two orientations with the highest Q_m (60° with 8750 and 90° with 8736) deliver the lowest $V_\pi L$ (1.303 and 0.979 V·cm). This strong correlation suggests that in our non-suspended LTOI devices, the acoustic quality factor is the dominant factor governing AO modulation efficiency, outweighing the orientation-dependent variations in the coupling factor Γ.

3. **The k^2–Q_m inverse relationship is consistent with SAW theory.** The data reveal that orientations with high k^2 (e.g., −30° with ~0.013) exhibit lower Q_m (3713), and vice versa. This is a well-known trade-off in SAW resonator engineering: strong piezoelectric coupling is accompanied by piezoelectric stiffening, which enhances acoustic radiation into the substrate and increases scattering losses, thereby degrading Q_m[3, 5, 6]. In the context of AO modulators, this trade-off translates into a bandwidth–efficiency design choice: high k^2 favors wide modulation bandwidth, while high Q_m favors low $V_\pi L$.

4. **The 30° orientation: mode suppression.** The effective k^2 for the R0 mode approaches zero at $\theta = 30°$ due to the transformation properties of the piezoelectric tensor (see Supplementary Note S1). The measured $V_\pi L$ = 4.542 V·cm at this angle likely originates from residual excitation of other weakly coupled acoustic modes.

Conclusion: For non-suspended LTOI AO modulators, the modulation efficiency is primarily limited by Q_m. Device optimization should therefore prioritize maximizing the acoustic quality factor through crystal orientation selection and cavity design. The optimal propagation direction for the R0 mode is along the crystal Z-axis ($\theta = 90°$), which provides the highest Q_m (8736) and consequently the lowest $V_\pi L$ (0.979 V·cm). Future improvements may focus on reducing fabrication-induced deviations to bring the experimental coupling factor closer to the simulated optimum, thereby further enhancing modulation efficiency.

Supplementary Note S5. Optical-cavity characterization

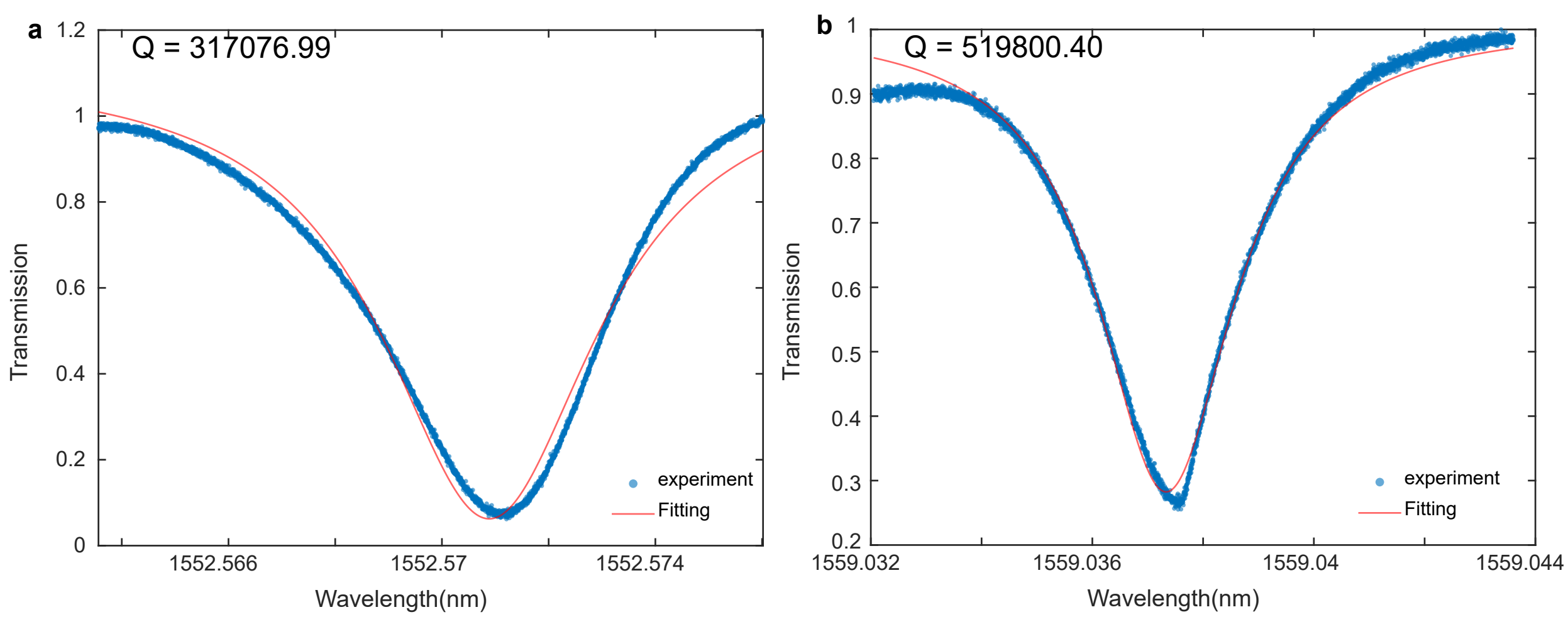

FIG. S8. **Measurement of the racetrack cavity.** (a) Transmission of racetrack cavity used in the low frequency AOM. (b) Transmission of racetrack cavity used in the high frequency AOM.

Based on the fabricated optical racetrack resonators, wavelength scanning was performed to obtain the transmission spectra. Lorentzian fitting was applied to the measured resonance dips, as presented in Fig. S8(a) and Fig. S8(b) for the racetrack cavities corresponding to Fig. S4(b) and Fig. S4(d), respectively. The extracted loaded quality factors (Q_L) were 3.17×10^5 and 5.20×10^5.

During the AO modulation measurements (Main text Figs. 4 and 5), the recorded optical powers detected by the PD were 0.13 mW (low frequency device) and 0.08 mW (high frequency device), respectively.

Supplementary Note S6. Theoretical analysis of modulation sidebands in the optical spectrum

To further elucidate the origin of the multiple resonance dips observed in the optical transmission spectrum (Fig. 5d of the main text), we provide a theoretical description of the acousto-optic (AO) modulation process. When the AO modulator is driven by a strong radio-frequency (RF) signal, the optical carrier at frequency f_{opt} is phase-modulated by the acoustic field at frequency f_a. The modulated electric field $E(t)$ can be expressed using the Jacobi-Anger expansion:

$$E(t) = E_0 e^{i\omega_0 t} \sum_{n=-\infty}^{\infty} J_n(\beta) e^{in\Omega t} \tag{S14}$$

where $\omega_0 = 2\pi f_{opt}$ is the optical carrier angular frequency, $\Omega = 2\pi f_a$ is the acoustic angular frequency, J_n denotes the n-th order Bessel function of the first kind, and β represents the modulation depth. The modulation depth β is proportional to the square root of the applied RF power. In a high-Q optical resonator ($Q_L \approx 5.2 \times 10^5$), these generated sidebands ($f_{opt} \pm n \cdot f_a$) align with the cavity resonance modes during the laser wavelength scanning process. As the RF power increases, the energy transfer from the optical carrier to these sidebands becomes more efficient, as determined by the power distribution $P_n \propto |J_n(\beta)|^2$. Consequently, the sideband dips in the transmission spectrum become deeper and more numerous. This behavior is consistent with the measured RF-induced modulation in the non-suspended LTOI resonator.

Supplementary Note S7. Simulation of a ring-shaped acoustic resonator

To demonstrate the feasibility of device miniaturization, we performed finite element method (FEM) simulations of a ring-shaped acoustic resonator. As illustrated in Fig. S9, ring-shaped interdigital transducers (IDTs) were designed to excite acoustic waves that conform to a circular optical micro-ring. The simulated admittance curve, shown in Fig. S9(a), indicates the effective excitation of acoustic modes. The displacement field distribution at the resonance frequency of 0.438 GHz is presented in Fig. S9(b). The results confirm that the acoustic energy can be sufficiently confined within the ring structure, suggesting that the device footprint can be significantly reduced compared to the racetrack topology while maintaining sufficient overlap for modulation.

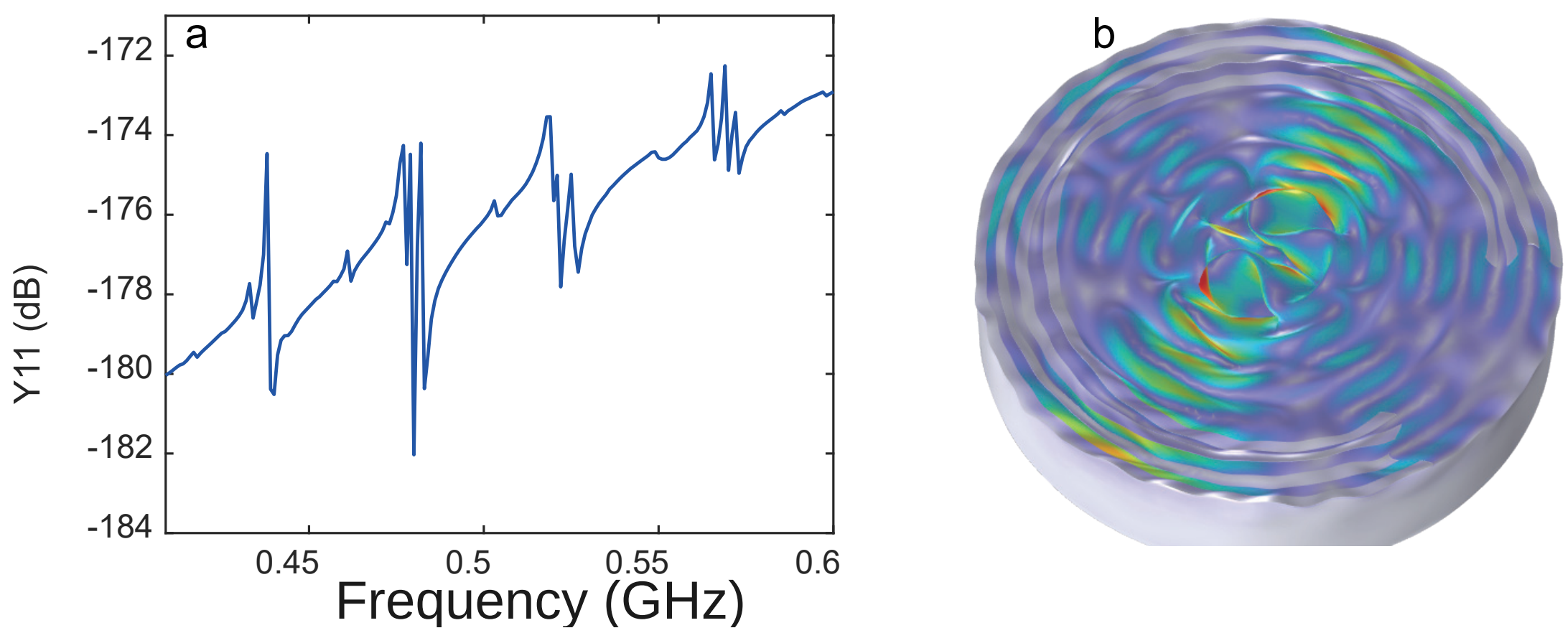

FIG. S9. **Simulation of ring-shaped acoustic resonator.** (a) Calculated admittance curve (Y_{11}) obtained from FEM simulation of the ring-shaped acoustic resonator. (b) Displacement field distribution of the acoustic mode at a frequency of 0.438 GHz.

Supplementary Note S8. Theoretical analysis of detuning dependence

To understand the dependence of acousto-optic modulation efficiency on cavity detuning, we consider the theory of cavity optomechanics. The optomechanical interaction induces an additional optical damping rate (Γ_{opt}) on the mechanical mode, which modifies the intrinsic mechanical damping rate. This optical damping rate is a function of the detuning $\Delta = \omega_{cavity} - \omega_{laser}$ and is given by [7]:

$$\Gamma_{opt}(\Delta) = g_0^2 N \left(\frac{\kappa}{(\Delta - \Omega_m)^2 + (\kappa/2)^2} - \frac{\kappa}{(\Delta + \Omega_m)^2 + (\kappa/2)^2} \right) \tag{S15}$$

where g_0 is the vacuum optomechanical coupling rate, N is the intracavity photon number, κ is the cavity decay rate, and Ω_m is the mechanical frequency.

The sign of Γ_{opt} determines whether the optomechanical interaction amplifies or damps the mechanical motion:

- **Blue Detuning ($\Delta < 0$):** The second term dominates, leading to $\Gamma_{opt} < 0$. This negative damping indicates optical gain for the mechanical mode, where pump photons are scattered to higher frequencies (anti-Stokes process) while absorbing phonons from the mechanical oscillator. This amplifies the acoustic oscillation, thereby enhancing the modulation efficiency observed in the S_{21} measurements.

- **Red Detuning ($\Delta > 0$):** The first term dominates, leading to $\Gamma_{opt} > 0$. This results in additional damping (cooling) of the mechanical motion through Stokes scattering, which suppresses the modulation signal.

Supplementary Note S9. Acoustic-reflector and waveguide-etch optimization

To quantitatively evaluate the enhancement brought by these reflection gratings, we calculated the modulated effective refractive index (Δn). The complete calculation of Δn, which accounts for the photo-elastic effect, electro-optic effect, and moving boundary effect ($\Delta n = \Delta n_{pe} + \Delta n_{eo} + \Delta n_{mb}$), was performed using the acousto-optic overlap integrals and the corresponding physical tensors detailed in **Supplementary Note S3**.

Finite-element method (FEM) simulations were conducted to compare the performance of the devices with and without the acoustic gratings. The corresponding refractive index change as a function of the driving frequency is shown in Fig. S10.

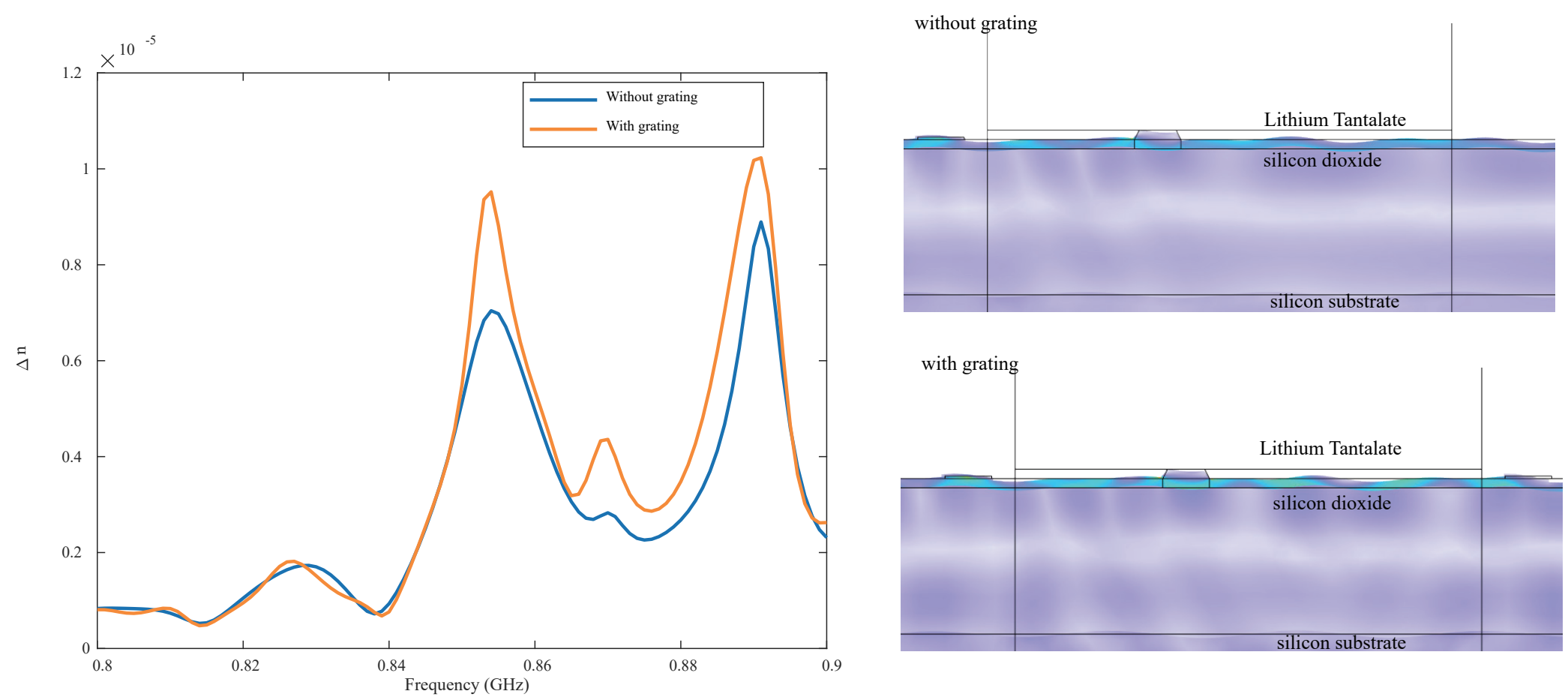

FIG. S10. **Quantitative analysis of the acoustic reflection gratings.** Simulated refractive index change (Δn) as a function of frequency for devices with and without the acoustic reflectors. The numerical results demonstrate that the implementation of the reflection gratings (with an identical electrode linewidth to the IDT and a 3Λ spacing) effectively confines the acoustic energy, enhancing the peak refractive index modulation by a factor of 1.1.

As illustrated by the simulation results, the maximum modulated refractive index (Δn) is enhanced by a factor of 1.1 (reaching approximately 1.0×10^{-5}) compared to the baseline device without gratings. This quantitative analysis confirms that the specific grating design successfully concentrates the acoustic energy, thereby improving the overall acousto-optic modulation efficiency.

To justify the structural design of the acousto-optic modulator, we systematically investigated the impact of the waveguide etching depth on both the optical propagation loss and the electro-acoustic conversion efficiency. The device utilizes a 600-nm thin-film lithium tantalate platform, and the etching depth dictates the geometry of the rib waveguide and the adjacent slab region.

Finite-element method (FEM) simulations were conducted to evaluate various etching depths. The selection of this depth involves a fundamental trade-off. On one hand, a deeper etch provides stronger lateral confinement for the optical mode within the rib waveguide, thereby reducing the optical propagation loss. On the other hand, a larger structural discontinuity at the etched step boundary exacerbates acoustic reflections and scattering when the surface acoustic wave propagates from the interdigital transducer (IDT) into the waveguide region. This scattering generates spurious modes and degrades the effective overlap between the acoustic field and the optical mode, consequently lowering the overall modulation efficiency.

The simulated refractive index change (Δn) and optical loss as a function of the etching depth are presented in Fig. S11.

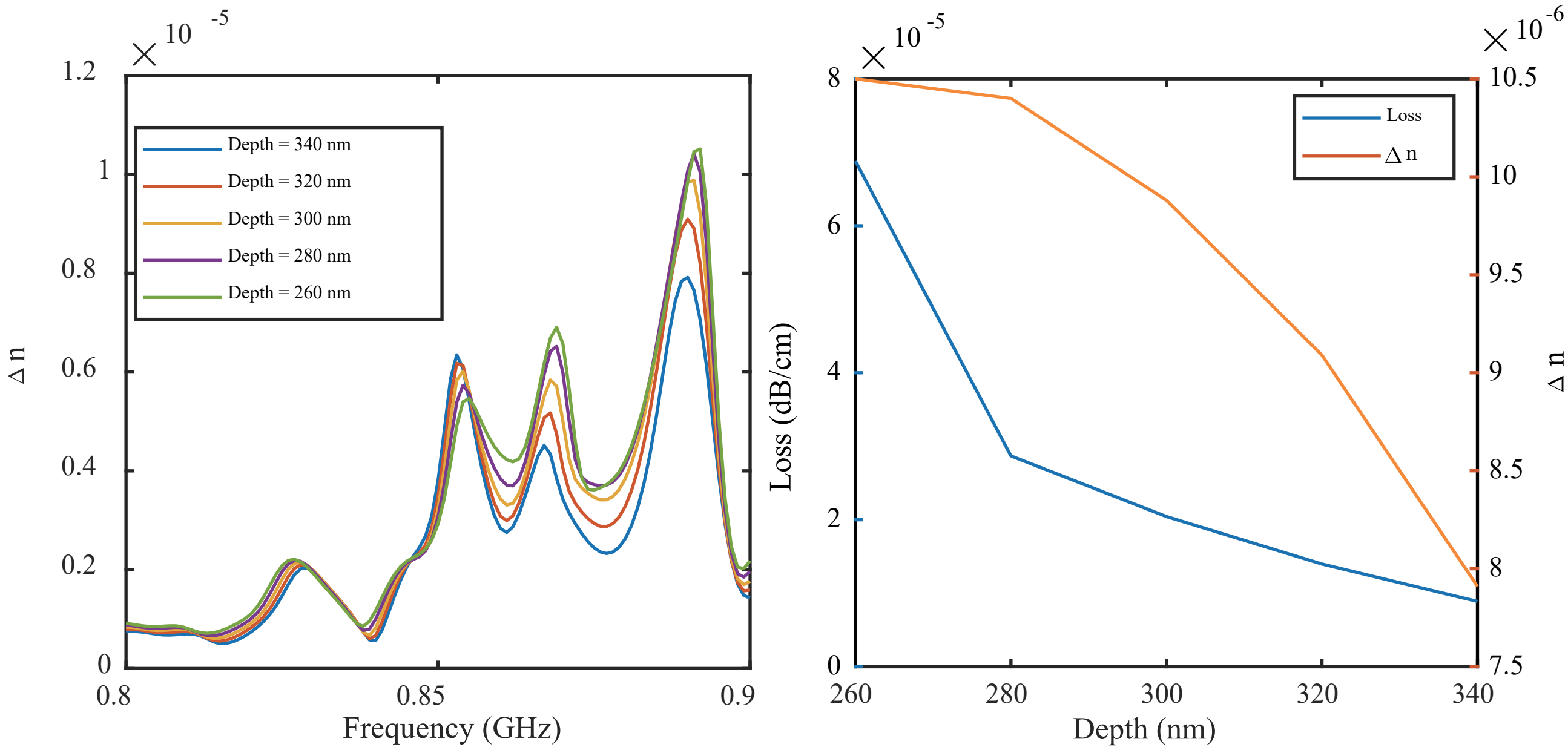

FIG. S11. **Quantitative analysis of the waveguide etching depth.** Simulated refractive index change (Δn) and optical propagation loss as a function of the etching depth. The numerical results illustrate a clear trade-off: deeper etching improves optical confinement (lowering the propagation loss) but introduces acoustic scattering at the step boundaries, which degrades the peak refractive index modulation. An etching depth of 300 nm was selected as the optimal balance.

As illustrated by the simulation results, the optical propagation loss consistently decreases with increasing etch depth, while the peak refractive-index modulation (Δn) begins to decrease. An etching depth of 300 nm, leaving a 300-nm slab, was therefore selected as a balance between the simulated optical loss and electro-acoustic response.

Supplementary Note S10. Temperature-dependent R0 resonance measurements of LT and LN reference acoustic structures

Acoustic resonators with identical design parameters were fabricated on LT and LN wafers. A thermoelectric cooler was used to control the device temperature at 20, 30, 40, and 50 °C. At each temperature, the R0 resonance was extracted from the minimum of the measured S_{11} spectrum using the same measurement and fitting procedure for both materials.

$$\mathrm{TCF} = \frac{1}{f_\mathrm{r}(T_0)} \frac{\mathrm{d}f_\mathrm{r}}{\mathrm{d}T} \times 10^6 \quad \mathrm{ppm/^\circ C}, \tag{S16}$$

where $T_0 = 25$ °C. Linear fitting gives TCF values of −25.13 ppm/°C for the measured LT resonator and −62.94 ppm/°C for the measured LN resonator. Within the tested 20–50 °C range, the R0 resonance of the LT structure exhibited the smaller temperature-induced frequency shift (Fig. S12).

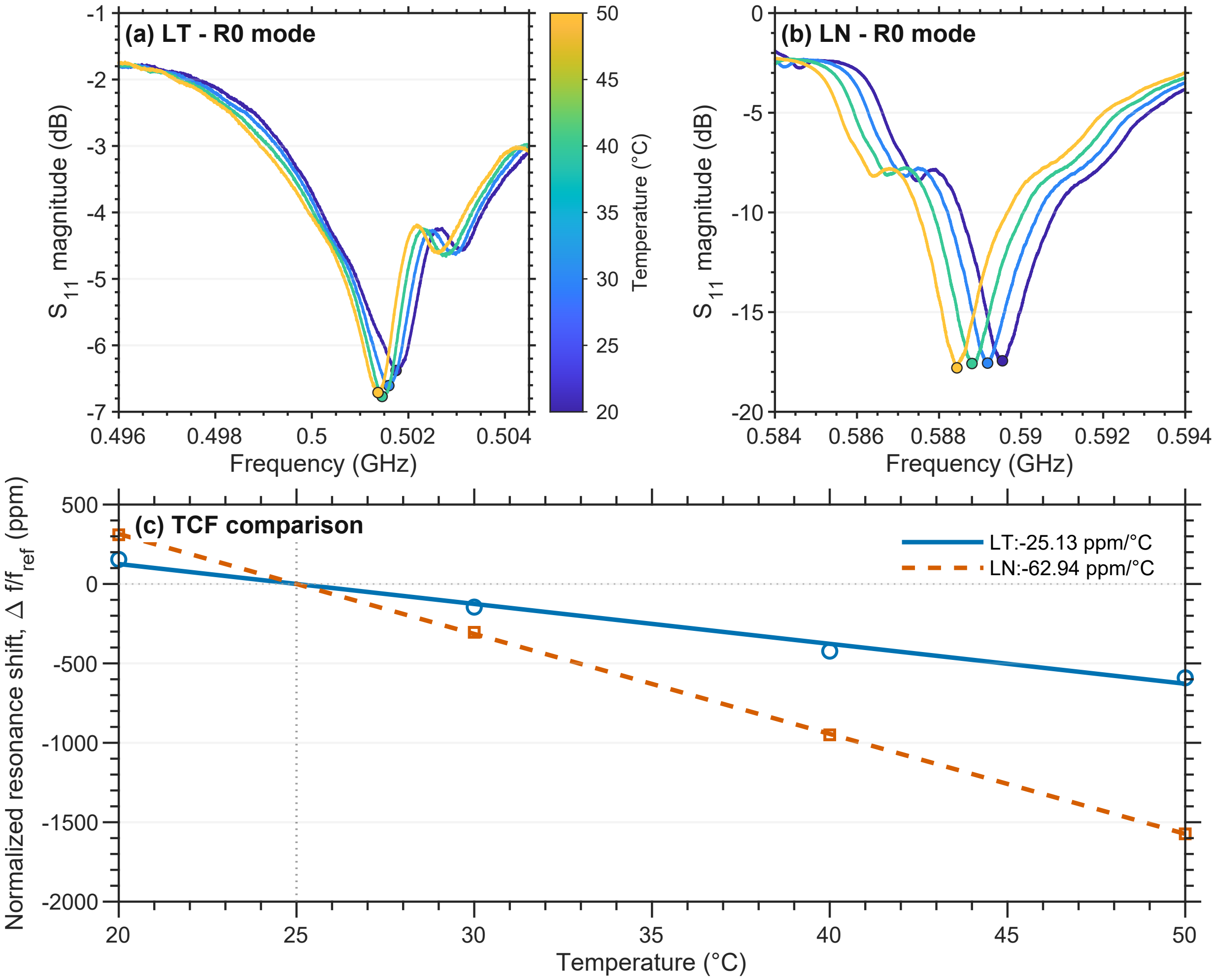

FIG. S12. **Temperature-dependent R0 resonance measurements of the LT and LN reference acoustic structures.** (a,b) Measured S_{11} spectra from 20 to 50 °C. (c) Normalized resonance-frequency shifts and linear fits; the extracted TCF values are shown in the panel.

Supplementary Note S11. RF-driven detected-output stability of the LTOI acousto-optic Mach–Zehnder interferometer

Previous studies have reported weaker photorefractive effects in LT than in LN [8]. To evaluate long-term operation of our device, the detected output of the LTOI acousto-optic Mach–Zehnder interferometer was recorded for 30 min under a continuous 0.858-GHz, 20-dBm RF drive and a 10-dBm input optical power. Figure S13 shows the raw detected-output record together with its 10-s average, both expressed relative to the initial level. The final 1.5-min window retained 79.69% of the initial detected output, corresponding to a change of approximately −0.99 dB.

The output therefore remained within approximately 1 dB over 30 min under simultaneous optical and RF excitation, supporting stable long-term operation of the LTOI acousto-optic modulator. This behavior is consistent with the lower photorefractive response reported for LT and contrasts with the stronger long-term drift reported for thin-film LN MZI devices in Ref. [8].

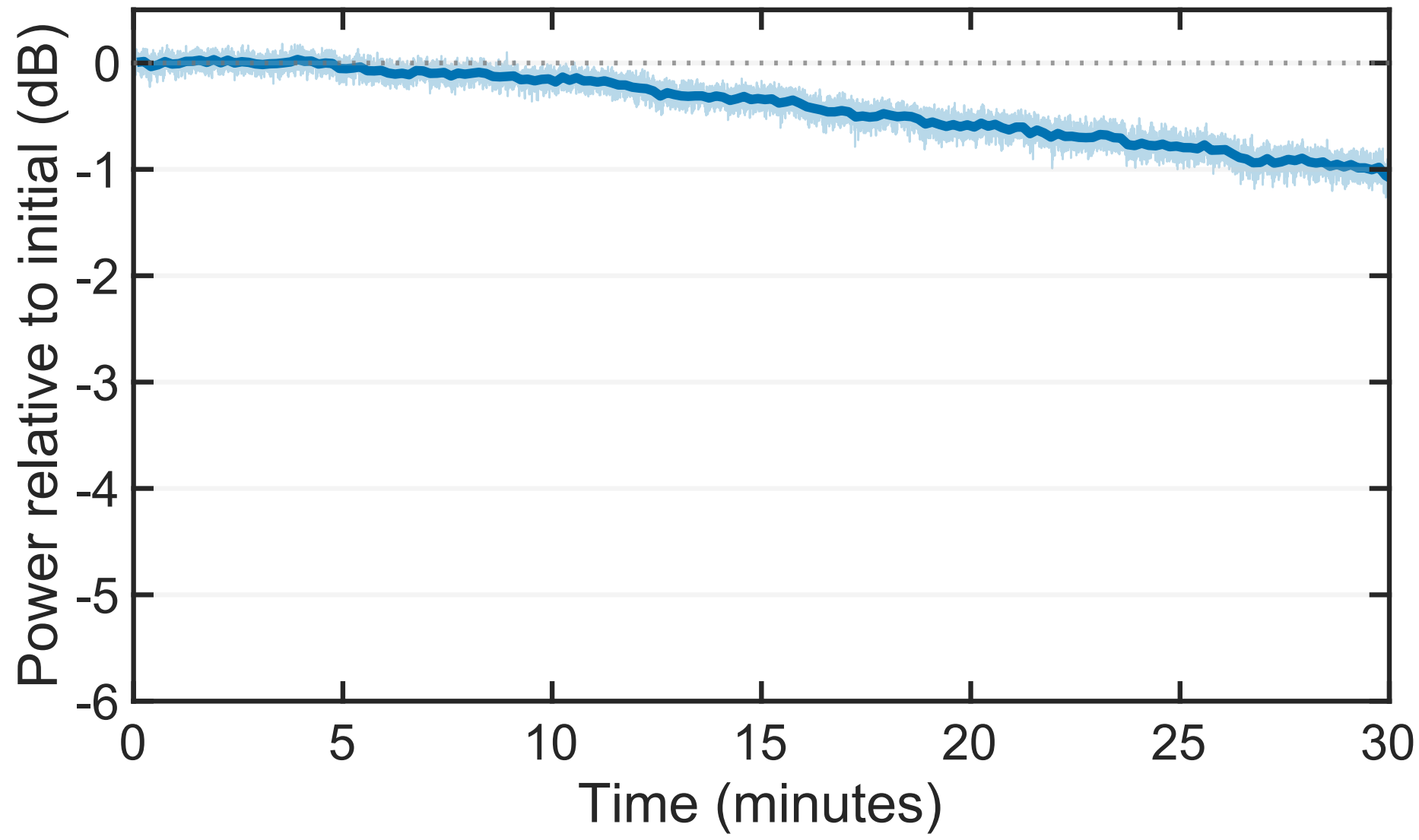

FIG. S13. **Thirty-minute RF-driven output-stability measurement of the LTOI acousto-optic MZI.** The light-blue trace shows the raw detected output and the dark-blue line shows the 10-s average under a 0.858-GHz, 20-dBm RF drive and a 10-dBm input optical power.

[1] Chang, S., Rogacheva, N. & Chou, C. Analysis of methods for determining electromechanical coupling coefficients of piezoelectric elements. *IEEE transactions on ultrasonics, ferroelectrics, and frequency control* **42**, 630–640 (2002).
[2] Smith, R. Elastic, piezoelectric, and dielectric properties of lithium tantalate. *Applied Physics Letters* **11**, 146–148 (1967).
[3] Hashimoto, K.-y. & Hashimoto, K.-Y. *Surface acoustic wave devices in telecommunications*, vol. 116 (Springer, 2000).
[4] Shao, L. *et al.* Microwave-to-optical conversion using lithium niobate thin-film acoustic resonators. *Optica* **6**, 1498–1505 (2019).
[5] Takai, T. *et al.* Ihp saw technology and its application to microacoustic components. In *2017 IEEE International Ultrasonics Symposium (IUS)*, 1–8 (IEEE, 2017).
[6] Tanaka, S., Guo, Y. & Kadota, M. Evolution of saw and baw devices using thin litao 3 and linbo 3. In *2024 IEEE MTT-S International Conference on Microwave Acoustics & Mechanics (IC-MAM)*, 169–172 (IEEE, 2024).
[7] Jiang, W. *et al.* Lithium niobate piezo-optomechanical crystals. *Optica* **6**, 845–853 (2019).
[8] Powell, K. *et al.* A sub-volt near-IR lithium tantalate electro-optic modulator. *APL Photonics* **10**, 091303 (2025).
